# Years of Life Lost to COVID‐19 and Related Mortality Indicators: An Illustration in 30 Countries

**DOI:** 10.1002/bimj.202300386

**Published:** 2024-07-13

**Authors:** Valentin Rousson, Isabella Locatelli

**Affiliations:** ^1^ Center for Primary Care and Public Health (Unisanté) University of Lausanne Lausanne Switzerland

**Keywords:** COVID‐19, excess mortality, life expectancy loss, weighted standardized mortality rate, years of life lost

## Abstract

The concept of (potential) years of life lost is a measure of premature mortality that can be used to compare the impacts of different specific causes of death. However, interpreting a given number of years of life lost at face value is more problematic because of the lack of a sensible reference value. In this paper, we propose three denominators to divide an excess years of life lost, thus obtaining three indicators, called *average life lost*, *increase of life lost*, and *proportion of life lost*, which should facilitate interpretation and comparisons. We study the links between these three indicators and classical mortality indicators, such as life expectancy and standardized mortality rate, introduce the concept of *weighted standardized mortality rate*, and calculate them in 30 countries to assess the impact of COVID‐19 on mortality in the year 2020. Using any of the three indicators, a significant excess loss is found for both genders in 18 of the 30 countries.

## 1 Introduction

The concept of (potential) years of life lost has been introduced as a measure of premature mortality, where one estimates for each death how long the person would have lived had he/she not died prematurely (Gardner and Sanborn 1990). It is used, for example, to compare the impacts of different specific causes of death (Murray and Lopez 1996). More recently, it has been used to evaluate the impact of COVID‐19 on mortality (Arolas et al. 2021), where the authors calculated a total of 20.5 million years lost to COVID‐19 across 81 countries (up to January 6, 2021). To make this calculation, the authors used two different approaches, “one based on COVID‐19 attributable deaths, and, for selected countries, one based on estimated excess deaths comparing recent mortality levels to an estimated baseline.” However, besides the fact that it is delicate to interpret a total without a denominator, calculating years of life lost based on the first approach is even more problematic since “whatever age at which a person dies, there are always additional years that the person might have lived” (Marshall 2004). As a consequence, it cannot be compared with zero, and it becomes especially difficult to judge how impressive such a total is. This is why Rousson and Locatelli (2022) followed the second approach to calculate the total years of life lost to COVID‐19 in 12 European countries in the year 2020 and proposed to divide this total by the size of the population concerned to obtain an average life lost to COVID‐19 in these countries. In the present paper, we further investigate and extend this concept by proposing three alternative denominators to divide the total years of life lost to COVID‐19, and by studying how these concepts are related to classical mortality indicators, such as life expectancy and standardized mortality rate (SMR). We also introduce the concept of weighted standardized mortality rate (WSMR) and compare the results obtained using the different indicators to assess the impact of COVID‐19 on mortality in 2020 in 30 countries.

## 2 Indicators Based on Years of Life Lost

In what follows, we consider mortality and demographic data for a given country and gender in a given year y (or a given period). Years of life lost (YLL) to a specific disease, such as COVID‐19 that we are considering throughout this article, are often calculated as

$$\text{YLL}=\sum_{x}c_{x}^{y}r_{x}^{y},$$

where $c_{x}^{y}$ is the number of deaths aged x attributed to this disease and $r_{x}^{y}$ is an expected number of remaining years still to be lived for an individual aged x that year. Here and below, sums are taken over the whole age range considered, typically for x=0,…,110, but they can also be restricted to a specific age group of interest. It is also possible to combine results for men and women in a given country by considering a sum over the different ages and the two genders. In addition to the fact that it cannot be negative, which makes it difficult to compare with any sensible value, as mentioned in the Introduction, this type of indicator takes no account of the fact that a person who died of COVID‐19 may have died of another cause had he or she not contracted COVID‐19, nor of the possible indirect effect of COVID‐19 on mortality, such as a death due to a preventive operation delayed as a result of COVID‐19, for example. This is why the overall effect of COVID‐19 on mortality has often been calculated via an excess of all‐cause mortality, not via specific mortality (Beaney et al. 2020). In this context, one may calculate an *excess of life lost* (ELL) as

$$\text{ELL}=\sum_{x}\left(d_{x}^{y}-f_{x}^{y}\right)r_{x}^{y},$$

where $d_{x}^{y}$ is the number of all‐cause deaths aged x, and $f_{x}^{y}$ is an expected number of all‐cause deaths aged x, so that $d_{x}^{y}-f_{x}^{y}$ represents the excess deaths aged x that year, compared to a “normal” year. In a context where a single exceptional event affecting mortality has occurred during the year, such as COVID‐19 in year y=2020, ELL can be interpreted as the total years of life lost to COVID‐19 in the population concerned. Note that ELL corresponds to the second approach to evaluate the impact of COVID‐19 on mortality mentioned in the Introduction, while YLL would correspond to the first approach.

Even though ELL, contrary to YLL, can take on positive or negative values, so that 0 is now a natural reference value indicating no excess loss, it remains difficult to interpret such a total at face value without further reference. In this paper, we propose three possible denominators to divide this quantity, namely,

$$D_{1}=\sum_{x}\left(n_{x}^{y}-f_{x}^{y}\right),\;D_{2}=\sum_{x}f_{x}^{y}r_{x}^{y}\;\text{and}\;D_{3}=\sum_{x}\left(n_{x}^{y}-f_{x}^{y}\right)r_{x}^{y},$$

where $n_{x}^{y}$ is the size of the population aged x that year (or the average population size over the year). Using the first one, an excess of life lost is divided by the number of individuals in the population who would still be alive without any excess of mortality, that is, the population of potential survivors. One obtains an *average life lost* (ALL) as

$$\text{ALL}=\text{ELL}/D_{1},$$

expressed in years (or in another unit of time). Using the second one, an excess of life lost is divided by what was expected to be lost. One obtains an *increase of life lost* (ILL) as

$$\text{ILL}=\text{ELL}/D_{2},$$

expressed in percent. Using the third one, the excess of life lost is divided by its maximum possible value, which would occur if everybody died. One obtains a *proportion of life lost* (PLL) as

$$\text{PLL}=\text{ELL}/D_{3},$$

also expressed in percent, which cannot exceed 100% (contrary to ILL). Note that the third one corresponds to a calculation suggested by Goldstein and Lee (2020) based on a scenario for the USA in 2020, whereas the first one is a slight modification of the denominator $\sum_{x}n_{x}^{y}$ used in Rousson and Locatelli (2022). This modification was carried out in order to obtain a more sensible value for ALL in the worst‐case scenario, if everyone died, corresponding to the average remaining life of the population of potential survivors, but with almost no effect on the results in realistic situations such as those presented below.

Even when ALL, ILL, and PLL are calculated using official and exhaustive data from a given country, they are still subject to variability, especially in a small country, which is important to consider if one wishes to generalize the effects of COVID‐19 on mortality in another similar situation (Curtin and Klein 1995). For inference purposes, we shall consider that the $f_{x}^{y}$, $r_{x}^{y}$, and $n_{x}^{y}$ are all fixed (or with negligible variability) and that only the $d_{x}^{y}$ are random, for which a Poisson distribution will be assumed, such that $\text{Var}\left(d_{x}^{y}\right)=f_{x}^{y}$. It follows that

$$\begin{array}{ccc} \text{Var}(\text{ALL}) & = & \frac{\sum_{x}f_{x}^{y}\cdot {\left(r_{x}^{y}\right)}^{2}}{D_{1}^{2}},\;\text{Var}\left(\text{ILL}\right)=\frac{\sum_{x}f_{x}^{y}\cdot {\left(r_{x}^{y}\right)}^{2}}{D_{2}^{2}},\;\text{and}\\ \text{Var}(\text{PLL}) & = & \frac{\sum_{x}f_{x}^{y}\cdot {\left(r_{x}^{y}\right)}^{2}}{D_{3}^{2}}. \end{array}$$

Approximate 95% confidence intervals (95% CI) for the average, increase, and proportion of life lost, that one would get in an infinite similar population, are obtained as

$$\begin{array}{ccc} & & \text{ALL}\;\pm \;1.96\cdot \text{Var}{\left(\text{ALL}\right)}^{1/2},\;\text{ILL}\;\pm \;1.96\cdot \text{Var}{\left(\text{ILL}\right)}^{1/2},\;\text{and}\\ & & \text{PLL}\;\pm \;1.96\cdot \text{Var}{\left(\text{PLL}\right)}^{1/2}. \end{array}$$

In what follows, we consider a situation where a 95% CI does not include (and is lying on the right of) the value 0 as a significant excess loss. Note also that by construction, a significant excess loss with respect to one indicator (ALL, ILL, or PLL) implies a significant excess loss with respect to the other two.

There are different approaches to calculate an expected number of deaths $f_{x}^{y}$, with potentially different results, as illustrated in Locatelli and Rousson (2023), where the pros and cons of two main approaches are discussed. A simple one is a factual comparison with the previous year, such that

$$f_{x}^{y}=\left(d_{x}^{y-1}/n_{x}^{y-1}\right)n_{x}^{y}.$$

This was used in Rousson and Locatelli (2022), together with $r_{x}^{y}=e_{x}^{y-1}$, where $e_{x}^{y}$ denotes the life expectancy at age x in year y obtained from a period life table. These choices of $f_{x}^{y}$ and $r_{x}^{y}$ will be used in the next sections, although other choices could be made, for example, taking $f_{x}^{y}$ based on a statistical model to predict mortality in year y, or taking $r_{x}^{y}$ based on projected cohort (instead of period) life tables. Although it may provide more stable results, calculating $f_{x}^{y}$ on the basis of average mortality over the last 3 or 5 “normal years” is however not recommended in a context of decreasing mortality, as it would ignore human progress in this regard, so that excess mortality would be underestimated (e.g., Ferenci 2023).

## 3 Life Lost to COVID‐19 in 30 Countries in 2020

In this section, we calculate indicators ALL, ILL, and PLL in the year 2020 for men and women in 30 countries, using official statistics from the Human Mortality Database (HMD 2024). We included the 30 countries with available and completed mortality data for the year 2020 at the time of writing this article (last access: May 14, 2024), namely Australia (AUS), Belgium (BEL), Bulgaria (BUL), Canada (CAN), Chile (CHL), Croatia (CRO), Czechia (CZE), Denmark (DEN), Finland (FIN), France (FRA), Germany (GER), Hong Kong (HKG), Hungary (HUN), Iceland (ICE), Ireland (IRE), Italy (ITA), Japan (JAP), Lithuania (LIT), Luxembourg (LUX), the Netherlands (NED), Norway (NOR), New Zealand (NZL), Portugal (POR), Republic of Korea (KOR), Spain (SPA), Sweden (SWE), Switzerland (SWI), Taiwan (TWN), the United Kingdom (UK), and the USA. For each country and gender and each age x, we had the numbers of deaths in 2019 and 2020, $d_{x}^{2019}$ and $d_{x}^{2020}$, the remaining life expectancies in 2019 $e_{x}^{2019}$, and the average population sizes in 2019 and 2020, $n_{x}^{2019}$ and $n_{x}^{2020}$, where $n_{x}^{2019}$ was obtained as the average of population sizes on the January 1, 2019 and 2020, and $n_{x}^{2020}$ as the average of population sizes on the January 1, 2020 and 2021.

**FIGURE 1:**
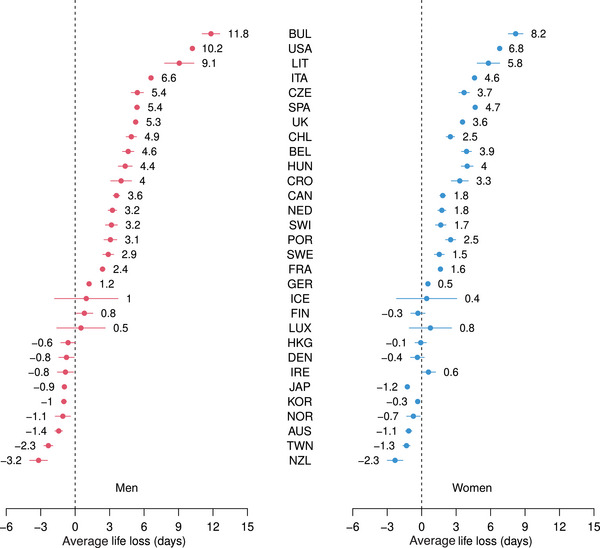
Average life loss for men and women of 30 countries in 2020, together with 95% CI.

**FIGURE 2:**
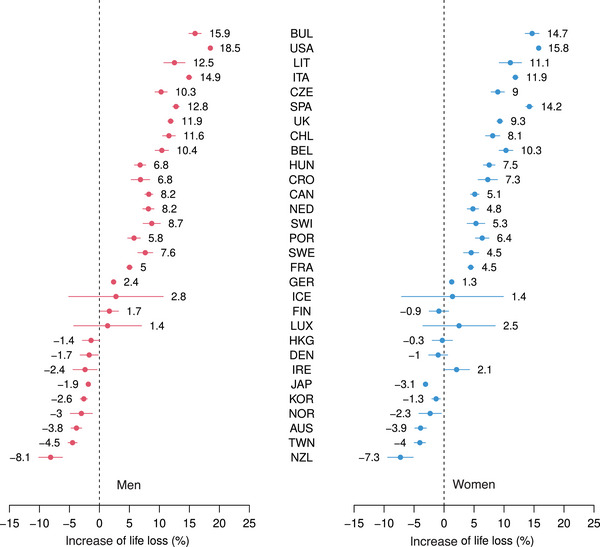
Increase of life lost for men and women of 30 countries in 2020, together with 95% CI.

**FIGURE 3:**
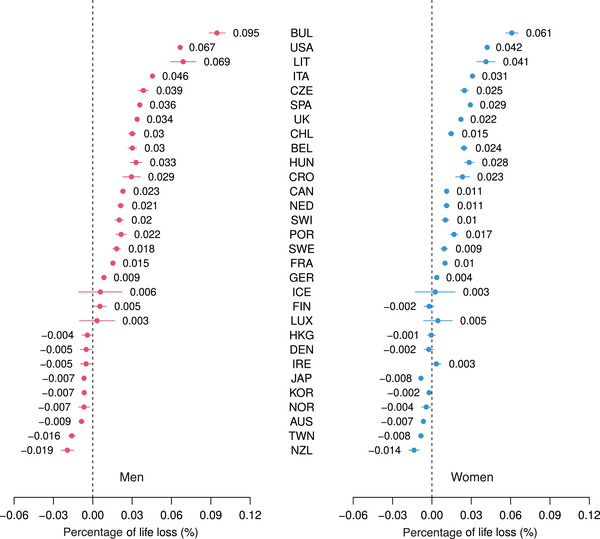
Proportion of life lost for men and women of 30 countries in 2020, together with 95% CI.

Results for the three indicators, together with 95% CI, can be seen in Figures 1, 2, 3, while Table 1 contains all results for ELL, ALL, ILL, and PLL, by gender and for men and women combined. Figures 1S–3S from the Supporting Information show a similar analysis, where confidence intervals have been calculated from 500 bootstrap replications of the original data with the same age distribution, with almost identical results. In all figures and tables, the 30 countries are ranked according to values of ALL for men (from the largest to the smallest). Bulgaria is found on the top of this ranking, followed by the USA and Lithuania. At the bottom of this ranking, we find New Zealand, Taiwan, and Australia, countries little or not affected by COVID‐19 in 2020, where losses were negative, thus corresponding to gains, as could be expected in a context of continuous human progress (i.e., in the absence of a pandemic), achieved by preventing and delaying deaths each year. Note that CI was logically much wider for small countries, such as Iceland or Luxembourg, than for large countries, such as the USA, where the width of CI was almost 0. While losses were consistently higher for men than for women, we had a significant excess loss in 18 of the 30 countries for both men and women. As noted by Rousson and Locatelli (2022), the natural scale to express an average life loss would be “days” rather than “years,” as shown in Figure 1 and Table 1. For example, while a total of 4530.1 thousand years have been lost to COVID‐19 for men in the USA in 2020, this corresponds to an average of ALL=10.2 days lost per person in the population of potential survivors (i.e., the population that would have survived if there had been no COVID‐19). Compared to 2019, the increase of “life lost” was of ILL=18.5%. However, the “proportion of life” that was lost was (only) of PLL=0.067%, or 6.7‱. This is in line with the scenario calculated in August 2020 by Goldstein and Lee (2020) regarding life loss to COVID‐19 in the USA in 2020, who concluded that “this represents a loss of less than 1/1000th of the population's remaining years to live.” Note that the three indicators were highly correlated, the Spearman correlations among them calculated over the 30 countries being 0.98, 0.996, and 0.96 for men and 0.99, 0.998, and 0.98 for women.

**TABLE 1:** Excess of life lost (ELL in thousand years), average life lost (ALL in days), increase of life lost (ILL in percent), and proportion of life lost (PLL in ‱) for men and women (separately and combined) of 30 countries in 2020. Negative losses correspond to gains. Countries are ranked according to values of ALL for men (from the largest to the smallest).

	Men				Women				Total			
	ELL	ALL	ILL	PLL	ELL	ALL	ILL	PLL	ELL	ALL	ILL	PLL
	(kyr)	(days)	(%)	(‱)	(kyr)	(days)	(%)	(‱)	(kyr)	(days)	(%)	(‱)
BUL	106.9	11.8	15.9	9.5	78.9	8.2	14.7	6.1	185.8	9.9	15.4	7.7
USA	4530.1	10.2	18.5	6.7	3081.0	6.8	15.8	4.2	7611.1	8.5	17.3	5.4
LIT	32.0	9.1	12.5	6.9	23.3	5.8	11.1	4.1	55.3	7.3	11.9	5.4
ITA	517.9	6.6	14.9	4.6	379.9	4.6	11.9	3.1	897.8	5.6	13.5	3.8
CZE	76.2	5.4	10.3	3.9	53.5	3.7	9.0	2.5	129.7	4.5	9.7	3.1
SPA	339.3	5.4	12.8	3.6	305.1	4.7	14.2	2.9	644.5	5.0	13.4	3.2
UK	473.0	5.3	11.9	3.4	327.5	3.6	9.3	2.2	800.5	4.4	10.7	2.8
CHL	116.3	4.9	11.6	3.0	62.0	2.5	8.1	1.5	178.3	3.7	10.1	2.2
BEL	71.2	4.6	10.4	3.0	61.9	3.9	10.3	2.4	133.2	4.3	10.4	2.7
HUN	55.0	4.4	6.8	3.3	54.4	4.0	7.5	2.8	109.3	4.2	7.1	3.1
CRO	21.3	4.0	6.8	2.9	18.6	3.3	7.3	2.3	39.9	3.6	7.0	2.6
CAN	184.0	3.6	8.2	2.3	95.0	1.8	5.1	1.1	279.0	2.7	6.8	1.7
NED	76.3	3.2	8.2	2.1	41.8	1.8	4.8	1.1	118.1	2.5	6.5	1.6
SWI	36.8	3.2	8.7	2.0	19.6	1.7	5.3	1.0	56.4	2.4	7.1	1.5
POR	41.0	3.1	5.8	2.2	37.2	2.5	6.4	1.7	78.2	2.8	6.0	1.9
SWE	40.8	2.9	7.6	1.8	21.4	1.5	4.5	0.9	62.2	2.2	6.2	1.4
FRA	204.5	2.4	5.0	1.5	149.4	1.6	4.5	1.0	353.9	2.0	4.8	1.3
GER	134.6	1.2	2.4	0.9	62.1	0.5	1.3	0.4	196.6	0.9	1.9	0.6
ICE	0.5	1.0	2.8	0.6	0.2	0.4	1.4	0.3	0.7	0.7	2.1	0.4
FIN	5.9	0.8	1.7	0.5	−2.5	−0.3	−0.9	−0.2	3.5	0.2	0.5	0.2
LUX	0.4	0.5	1.4	0.3	0.6	0.8	2.5	0.5	1.1	0.6	1.9	0.4
HKG	−5.7	−0.6	−1.4	−0.4	−0.9	−0.1	−0.3	−0.1	−6.6	−0.3	−0.9	−0.2
DEN	−6.0	−0.8	−1.7	−0.5	−2.9	−0.4	−1.0	−0.2	−8.9	−0.6	−1.4	−0.4
IRE	−5.7	−0.8	−2.4	−0.5	4.1	0.6	2.1	0.3	−1.6	−0.1	−0.4	−0.1
JAP	−152.3	−0.9	−1.9	−0.7	−214.2	−1.2	−3.1	−0.8	−366.5	−1.1	−2.4	−0.8
KOR	−68.5	−1.0	−2.6	−0.7	−24.2	−0.3	−1.3	−0.2	−92.7	−0.7	−2.1	−0.4
NOR	−7.9	−1.1	−3.0	−0.7	−5.2	−0.7	−2.3	−0.4	−13.1	−0.9	−2.7	−0.6
AUS	−49.4	−1.4	−3.8	−0.9	−39.5	−1.1	−3.9	−0.7	−88.8	−1.3	−3.9	−0.8
TWN	−74.0	−2.3	−4.5	−1.6	−42.7	−1.3	−4.0	−0.8	−116.7	−1.8	−4.3	−1.2
NZL	−21.8	−3.2	−8.1	−1.9	−16.1	−2.3	−7.3	−1.4	−37.8	−2.7	−7.7	−1.6

**FIGURE 4:**
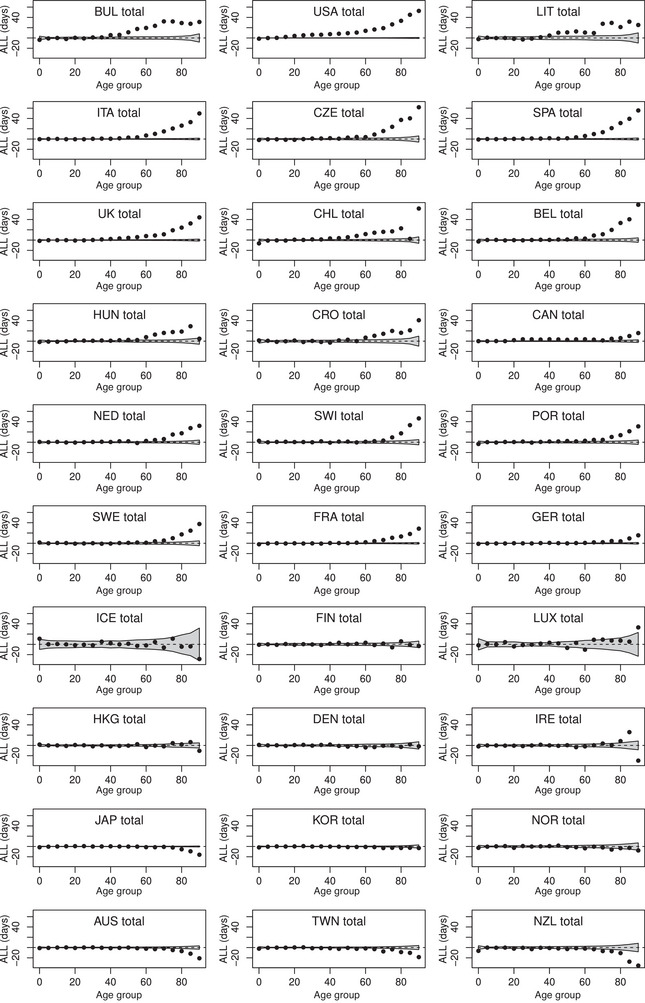
Average life lost (ALL in days) calculated in 5‐year age groups for men and women combined of 30 countries in 2020. Values outside the grey bands $\pm 1.96\cdot \text{Var}{\left(\text{ALL}\right)}^{1/2}$ are considered significant.

**FIGURE 5:**
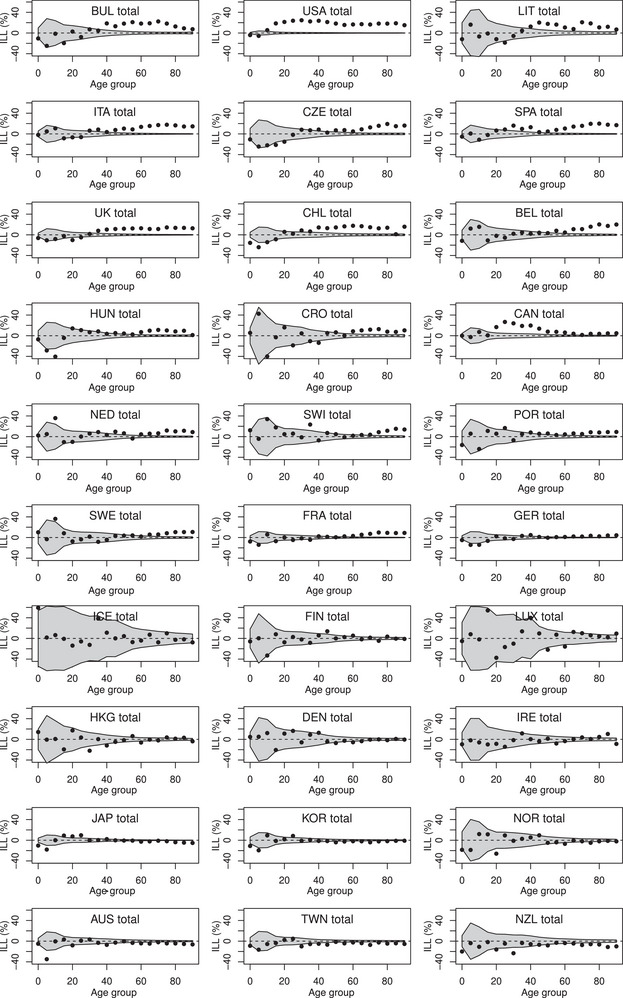
Increase of life lost (ILL in percent) calculated in 5‐year age groups for men and women combined of 30 countries in 2020. Values outside the grey bands $\pm 1.96\cdot \text{Var}{\left(\text{ILL}\right)}^{1/2}$ are considered significant.

**FIGURE 6:**
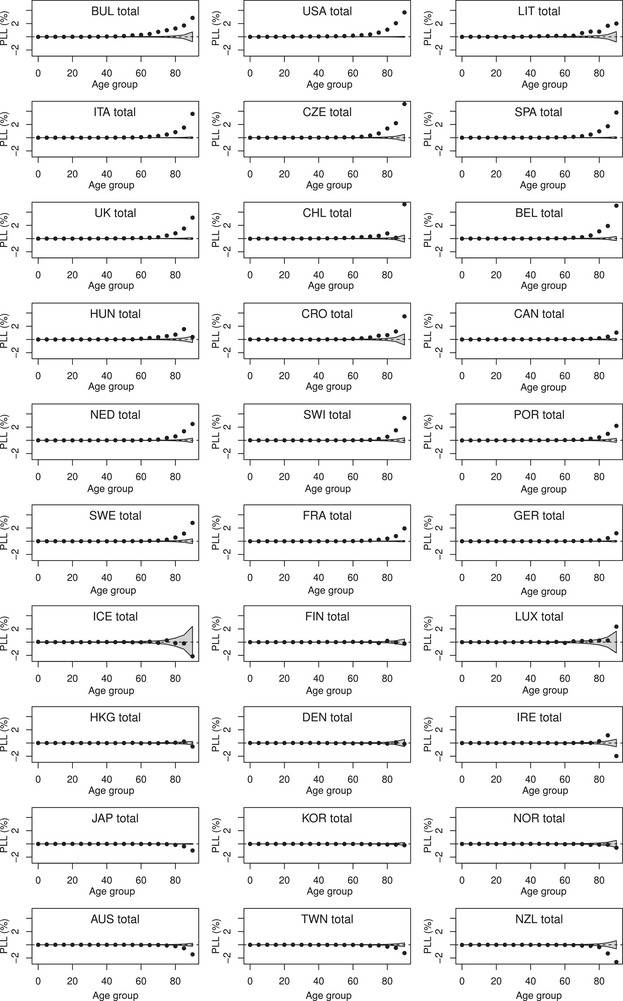
Proportion of life lost (PLL in percent) calculated in 5‐year age groups for men and women combined of 30 countries in 2020. Values outside the grey bands $\pm 1.96\cdot \text{Var}{\left(\text{PLL}\right)}^{1/2}$ are considered significant.

Figures 4, 5, 6 show ALL, ILL, and PLL calculated for 5‐year age groups (from 0–4 to 85–89, with a last age group 90–110) for men and women combined. In most countries with positive excess loss, ALL and PLL increased markedly with age, with ALL reaching more than 50 days and PLL being close to 4% in the oldest age group in the USA. In contrast, the increase of ILL with age was less evident, although a significant ILL remained equivalent to a significant ALL or PLL in any age group. Note that statistical uncertainty (represented by gray bands on these figures) appears higher at younger ages for ILL, and at older ages for ALL and PLL. This is in line with the variance formulae provided in the previous section, with the variance at a given age decreasing with the expected number of deaths for ILL, with population size for ALL and PLL. The Spearman correlation between ALL and PLL calculated over the 30 countries and the 19 age groups remained high at 0.993, while it was only 0.87 between ALL and ILL and 0.83 between ILL and PLL. Figures 4S–9S from the Supporting Information show similar analyses by age and gender.

## 4 Average Life Lost Versus Life Expectancy Loss

A well‐known indicator for summarizing mortality data in a given year is life expectancy at birth (Luy et al. 2019), which has also been used as an indicator of human progress, as witnessed by the statement of Wilmoth (1997) that “the dramatic rise in life expectancy during the past two or three centuries is arguably the greatest collective human achievement.” Life expectancies at birth (LE) in 2019 and 2020 in the 30 countries taken from the Human Mortality Database can be found in Table 2 (for men) and in Table 3 (for women). For example, male life expectancy in the USA was 76.5 years in 2019, falling to 74.3 years in 2020, corresponding to a loss of 2.2 years (26.4 months) or 2.9%.

**TABLE 2:** Life expectancies at birth (LE), standardized mortality rates (SMR), and weighted standardized mortality rates (WSMR) *for men* of 30 countries in 2019 and 2020. The reference year for standardization in SMR and WSMR is 2020. Weights in WSMR are taken according to life expectancies in 2019. Positive differences indicate a LE loss, respectively an increase of SMR or WSMR, in 2020 compared to 2019. Countries are ranked according to values of average life loss for men (as in Table 1).

	LE				SMR			WSMR		
	2019	2020	diff.	diff.	2019	2020	diff.	2019	2020	diff.
	(years)	(years)	(months)	(%)	(‰)	(‰)	(%)	(‰)	(‰)	(%)
BUL	71.6	70.0	19.2	2.2	16.7	19.6	17.6	5.9	6.8	15.9
USA	76.5	74.3	26.4	2.9	9.2	10.8	18.1	3.6	4.3	18.5
LIT	71.5	70.1	16.8	2.0	14.3	16.3	14.2	5.5	6.2	12.5
ITA	81.1	79.8	15.6	1.6	10.7	12.4	16.1	3.0	3.5	14.9
CZE	76.3	75.2	13.2	1.4	11.2	12.8	14.2	3.7	4.1	10.3
SPA	80.7	79.5	14.4	1.5	9.2	10.7	15.8	2.8	3.2	12.8
UK	79.6	78.4	14.4	1.5	9.3	10.5	13.8	2.8	3.2	11.9
CHL	77.4	76.1	15.6	1.7	6.8	7.7	13.8	2.6	2.9	11.6
BEL	79.6	78.5	13.2	1.4	9.5	10.9	15.0	2.9	3.2	10.4
HUN	73.0	72.3	8.4	1.0	13.6	14.7	8.1	4.8	5.2	6.8
CRO	75.4	74.7	8.4	0.9	13.1	14.3	9.1	4.3	4.6	6.8
CAN	80.2	79.4	9.6	1.0	8.0	8.5	5.7	2.8	3.0	8.2
NED	80.5	79.7	9.6	1.0	8.8	9.7	10.2	2.6	2.8	8.2
SWI	81.9	81.0	10.8	1.1	7.8	8.8	12.1	2.3	2.5	8.7
POR	79.0	78.4	7.2	0.8	11.7	12.4	6.7	3.7	3.9	5.8
SWE	81.3	80.6	8.4	0.9	8.6	9.5	9.8	2.4	2.5	7.6
FRA	79.8	79.2	7.2	0.8	9.6	10.3	8.0	3.0	3.2	5.0
GER	78.8	78.6	2.4	0.3	11.6	12.0	3.3	3.6	3.7	2.4
ICE	81.3	81.2	1.2	0.1	6.7	6.6	−2.7	2.1	2.2	2.8
FIN	79.2	79.0	2.4	0.3	10.2	10.2	0.3	3.3	3.3	1.7
LUX	80.0	79.8	2.4	0.3	7.1	7.6	6.0	2.3	2.4	1.4
HKG	82.1	82.3	−2.4	−0.2	8.3	8.2	−1.3	3.0	2.9	−1.4
DEN	79.4	79.6	−2.4	−0.3	9.7	9.7	−0.8	2.9	2.9	−1.7
IRE	80.4	80.6	−2.4	−0.2	6.8	6.8	−0.8	2.1	2.1	−2.4
JAP	81.4	81.6	−2.4	−0.2	12.1	11.8	−2.9	3.6	3.5	−1.9
KOR	80.3	80.6	−3.6	−0.4	6.6	6.4	−2.2	2.5	2.4	−2.6
NOR	81.2	81.5	−3.6	−0.4	7.6	7.4	−2.6	2.2	2.2	−3.0
AUS	81.2	81.6	−4.8	−0.5	7.0	6.7	−4.2	2.2	2.2	−3.8
TWN	77.5	78.0	−6.0	−0.6	9.0	8.7	−4.0	3.6	3.4	−4.5
NZL	80.0	80.8	−9.6	−1.0	7.3	6.7	−7.3	2.4	2.2	−8.1

**TABLE 3:** Life expectancies at birth (LE), standardized mortality rates (SMR), and weighted standardized mortality rates (WSMR) *for women* of 30 countries in 2019 and 2020. The reference year for standardization in SMR and WSMR is 2020. Weights in WSMR are taken according to life expectancies in 2019. Positive differences indicate a LE loss, respectively an increase of SMR or WSMR, in 2020 compared to 2019. Countries are ranked according to values of average life loss for men (as in Table 1).

	LE				SMR			WSMR		
	2019	2020	diff.	diff.	2019	2020	diff.	2019	2020	diff.
	(years)	(years)	(months)	(%)	(‰)	(‰)	(%)	(‰)	(‰)	(%)
BUL	78.7	77.5	14.4	1.5	14.7	16.4	12.1	4.1	4.7	14.7
USA	81.5	79.9	19.2	2.0	8.3	9.7	16.5	2.7	3.1	15.8
LIT	81.0	80.0	12.0	1.2	13.5	15.0	10.9	3.7	4.1	11.1
ITA	85.4	84.5	10.8	1.1	11.0	12.5	13.8	2.6	2.9	11.9
CZE	82.1	81.3	9.6	1.0	10.4	11.7	12.4	2.8	3.0	9.0
SPA	86.2	85.0	14.4	1.4	8.7	10.1	16.7	2.1	2.3	14.2
UK	83.3	82.4	10.8	1.1	9.0	10.0	11.2	2.4	2.6	9.3
CHL	82.6	81.8	9.6	1.0	5.9	6.4	9.3	1.8	1.9	8.1
BEL	84.0	83.1	10.8	1.1	9.6	11.1	15.3	2.4	2.6	10.3
HUN	79.7	79.0	8.4	0.9	13.3	14.2	7.3	3.8	4.0	7.5
CRO	81.4	80.8	7.2	0.7	12.9	13.9	8.1	3.2	3.4	7.3
CAN	84.4	83.9	6.0	0.6	7.5	7.8	4.3	2.2	2.3	5.1
NED	83.6	83.1	6.0	0.6	9.0	9.6	7.2	2.3	2.4	4.8
SWI	85.6	85.1	6.0	0.6	8.2	8.9	8.3	1.9	2.0	5.3
POR	84.8	84.2	7.2	0.7	10.5	11.4	8.4	2.6	2.8	6.4
SWE	84.7	84.3	4.8	0.5	8.8	9.5	7.6	2.1	2.2	4.5
FRA	85.6	85.2	4.8	0.5	9.1	9.7	6.7	2.2	2.3	4.5
GER	83.5	83.4	1.2	0.1	11.4	11.7	2.4	2.9	2.9	1.3
ICE	84.5	84.3	2.4	0.2	6.7	6.7	−0.2	1.8	1.8	1.4
FIN	84.5	84.6	−1.2	−0.1	9.8	9.8	0.2	2.4	2.4	−0.9
LUX	84.8	84.4	4.8	0.5	7.0	7.2	3.7	1.8	1.9	2.5
HKG	88.1	88.0	1.2	0.1	5.6	5.6	0.0	1.8	1.8	−0.3
DEN	83.4	83.5	−1.2	−0.1	9.2	9.1	−1.4	2.4	2.4	−1.0
IRE	84.4	84.1	3.6	0.4	6.1	6.2	2.2	1.6	1.7	2.1
JAP	87.4	87.7	−3.6	−0.3	11.0	10.5	−4.3	2.7	2.6	−3.1
KOR	86.3	86.5	−2.4	−0.2	5.5	5.4	−1.9	1.6	1.6	−1.3
NOR	84.7	84.9	−2.4	−0.2	7.9	7.7	−2.1	1.9	1.8	−2.3
AUS	85.3	85.7	−4.8	−0.5	6.3	6.0	−5.2	1.7	1.6	−3.9
TWN	83.8	84.2	−4.8	−0.5	6.3	6.0	−5.1	2.1	2.0	−4.0
NZL	83.7	84.5	−9.6	−1.0	6.7	6.2	−8.6	1.9	1.7	−7.3

At first glance, it may seem curious, or even dubious, that the above average life lost of 10.2 days reported for men in the USA corresponds to a loss of life expectancy of 2.2 years. But this becomes perfectly understandable once we recall that life expectancy at birth in 2020 refers to the average age at death that would be reached by a hypothetical cohort of individuals who would be born and spend their entire lives under the mortality conditions observed in 2020. Thus, the life expectancy loss of 2.2 years corresponds to what was lost on average by this hypothetical population of men, who would have spent their entire lives with a virulent COVID‐19, as it was in 2020 (rather than spending their entire lives in 2019). In contrast, the average life lost of 10.2 days corresponds to what the real (not hypothetical) population of men living in the USA lost in 2020. In fact, in all our examples, the life expectancy loss for the year 2020 could be approximately retrieved by multiplying the average life lost for the year 2020 by the life expectancy in 2019. For men in the USA, the average life lost of 10.2/365.25 years multiplied by the life expectancy of 76.5 gives 2.14 years, almost matching the life expectancy loss of 2.2 years.

Goldstein and Lee (2020) reminded us that “in the context of epidemic mortality, life expectancy at birth is a misleading indicator because it implicitly assumes the epidemic is experienced each year over and over again as a person gets older.” An average life lost might thus be a more relevant indicator for quantifying what has really been lost by a population in a given year than a life expectancy loss. But while their scales and meanings are very different, the two indicators were highly correlated in our examples, Spearman correlations calculated over the 30 countries are 0.98 for men and 0.99 for women.

## 5 Increase and Proportion of Life Lost Versus Standardized Mortality Rates

Another classical indicator for summarizing mortality data in a given year is an SMR, defined as

$$\text{SMR}=\sum_{x}\left(d_{x}^{y}/n_{x}^{y}\right)s_{x}/\sum_{x}s_{x},$$

where $s_{x}$ is the size of a reference population at age x. This indicator provides us with the proportion of persons that would have died in the reference population were they subject to the mortality rates $d_{x}^{y}/n_{x}^{y}$ observed in a given year y. While an SMR depends on the reference population that is used (Spiegelman and Marks 1966), this indicator is useful to compare mortality data among populations with different age structures. On the other hand, an SMR does consider equally the deaths occurring at a younger age and those occurring at an older age. An alternative indicator would be to consider a WSMR, defined as

$$\text{WSMR}=\sum_{x}\left(d_{x}^{y}/n_{x}^{y}\right)s_{x}w_{x}/\sum_{x}s_{x}w_{x},$$

where $w_{x}$ is an importance weight assigned to age x, which could typically be proportional to the number of remaining years still to be lived at age x (or years to be lived in good health or to some economic value attributed to those years, among other possibilities).

Table 2 (for men) and Table 3 (for women) provide SMR and WSMR calculated in years 2019 and 2020 for the 30 countries, using $s_{x}=n_{x}^{2020}$ and $w_{x}=e_{x}^{2019}$. For both genders in all countries, WSMR was much smaller than SMR. However, the percentage increase in 2020 compared to 2019 was roughly comparable for SMR and WSMR in most countries. As an example, for men in the USA, the SMR increased from 0.0092 to 0.0108, whereas the WSMR increased from 0.0036 to 0.0043, corresponding to an increase of 18.1% in the former case a 18.5% in the latter case.

Whereas we did not find any trace of a WSMR concept in the statistical or demographical literature, it is closely related to the concepts of ILL and PLL introduced above. One can readily check that

$$\text{ILL}=\frac{{\text{WSMR}}_{1}-{\text{WSMR}}_{0}}{{\text{WSMR}}_{0}}\;\text{and}\;\text{PLL}=\frac{{\text{WSMR}}_{1}-{\text{WSMR}}_{0}}{{\text{WSMR}}_{max}-{\text{WSMR}}_{0}},$$

where

$${\text{WSMR}}_{1}=\sum_{x}\left(d_{x}^{y}/n_{x}^{y}\right)n_{x}^{y}r_{x}^{y}/\sum_{x}n_{x}^{y}r_{x}^{y}$$

is the WMSR calculated in year y using $s_{x}=n_{x}^{y}$ and $w_{x}=r_{x}^{y}$, where

$${\text{WSMR}}_{0}=\sum_{x}\left(f_{x}^{y}/n_{x}^{y}\right)n_{x}^{y}r_{x}^{y}/\sum_{x}n_{x}^{y}r_{x}^{y}$$

is the expected WSMR that year (for a “normal” year), and where ${\text{WSMR}}_{\max}=1$ is the maximal possible value for a WSMR. Using $f_{x}^{y}=\left(d_{x}^{y-1}/n_{x}^{y-1}\right)n_{x}^{y}$, one has

$${\text{WSMR}}_{0}=\sum_{x}\left(d_{x}^{y-1}/n_{x}^{y-1}\right)n_{x}^{y}r_{x}^{y}/\sum_{x}n_{x}^{y}r_{x}^{y},$$

that is, this is the WSMR calculated in year y−1. The eighth and ninth columns in Tables 2 and 3 (for men and women) correspond to such ${\text{WSMR}}_{0}$ and ${\text{WSMR}}_{1}$ with y=2020 and $w_{x}=r_{x}^{y}=e_{x}^{y-1}$. One can also check that the last column in these tables corresponds to the values of ILL provided in Table 1.

## 6 Conclusions

In this paper, we have proposed three possible denominators to divide an excess of life lost, which should facilitate interpretation and comparisons, and we have calculated the three resulting indicators in 30 countries to assess the impact of COVID‐19 on mortality in 2020, where a significant excess loss has been found for both genders in 18 of them. Once data become fully available, it will be interesting to repeat the analysis for years 2021 and 2022 to see in which countries there is still (or newly) a significant excess loss. While the three indicators are expressed on different scales (days, percentage increase, proportion), they look at different aspects of the same reality, being evidently highly correlated. They could also be calculated in a year without any major event impacting mortality, where negative losses would be interpreted as gains, which would be useful for quantifying progress in terms of mortality for a real population.

The first indicator, referred to as an *average life lost*, turns a total number of years lost into an average, which is a classic move in statistics, providing the average number of years lost in a given year for a real population. In contrast, a life expectancy loss would provide the average number of years lost over a lifetime for a hypothetical population. As a result, an average life lost is generally much lower than a life expectancy loss, being typically expressed in days rather than in years, which may make the impact of COVID‐19 seem less dramatic. Although not a good metric for quantifying the impact on mortality of a punctual event for a real population, such as a pandemic, calculating a life expectancy loss could still be useful for estimating the potential damage of a potentially long‐lasting or recurrent event, such as air pollution or global warming. Even without referring to a real population, life expectancy remains a powerful indicator to describe the evolution of mortality over years and centuries.

The second indicator, referred to as an *increase of life lost*, expresses an increase of years lost as a percentage, that is, on a relative rather than an absolute scale, which may provide a somewhat different picture of the situation than the other two indicators, as illustrated when the analysis was carried out by age group. We have seen that this is equivalent to a percentage increase of some *WSMR*, where the weights are taking account of the fact that more years are lost for a death occurring at a younger age than at an older age. Of note, the increase of life lost appeared in our examples much greater than the life expectancy loss (expressed in percent), the former being, for example, 18.5% and the latter (only) 2.9% for men in the USA in 2020 (compared to 2019).

The third indicator, referred to as the *proportion of life lost*, expresses a total number of years lost as a proportion of what would have been lost in the worst‐case scenario, that is, if everyone in the population concerned had died. The third indicator, therefore, provides much lower values than the second, for example, a loss of (only) 0.067% for men in the USA in 2020, which led Goldstein and Lee (2020) to write about COVID‐19 that “it is possible to portray the epidemic as unimaginably large—the biggest killer in American history—or small, reducing our remaining life expectancy by <1 part in 1000.” While it is not our purpose here to conclude whether a loss of around 1/1000 is substantial or not, it will be interesting to compare this loss with those caused by other diseases or risk factors, such as cancer or tobacco, or by other past pandemics.

It should also be remembered that our indicators were calculated while stringent measures were taken in 2020 to reduce the impact of COVID‐19, and no one knows exactly how many lives would have been lost in the absence of such measures. Finally, our indicators only provide information on the impact of COVID‐19 on mortality, and not on other potential health damage it might have, as evidenced by the increase in the number of intensive care admissions over this period. Similar indicators measuring an “excess of life lost in good health” rather than just an “excess of life lost” could be defined, even though they would be more difficult to implement as they would require more information.

## Conflicts of Interest

The authors have declared no conflicts of interest.

## Open Research Badges

This article has earned an Open Data badge for making publicly available the digitally‐shareable data necessary to reproduce the reported results. The data is available in the Supporting Information section.

This article has earned an open data badge “**Reproducible Research**” for making publicly available the code necessary to reproduce the reported results. The results reported in this article could fully be reproduced.

## Supporting information

Supporting Information

Supporting Information

## Data Availability

The original data that support the findings of this study are openly available in the Human Mortality Database at www.mortality.org.
