# Supplementary material for: Years of Life Lost to COVID‐19 and Related Mortality Indicators: An Illustration in 30 Countries
Source: Biom J. 2024 Jul 13;66(5):e202300386. doi: 10.1002/bimj.202300386 (PMC12859533; doi:10.1002/bimj.202300386)
Supplement: Supplementary file 1 — Supporting Information [file BIMJ-66-e202300386-s002.zip › reproductibility/README.pdf]

Title of manuscript: "Years of life lost to COVID-19 and related mortality indicators: An illustration in 30 countries"

Authors of manuscript: Valentin Rousson & Isabella Locatelli

Authors of code: Valentin Rousson & Isabella Locatelli

Emails: valentin.rousson@unisante.ch;
isabella.locatelli@unisante.ch

This program reproduces Tables 1-3 and Figures 1-6 from this article, as well ad Figures 1S-9S from the Supporting Information, and display the Spearman correlations reported in Sections 3 and 4 of the article.

File "functions.R" contains functions used in "main.R"

To get these results, just run in R: > source("main.R")

The program will print correlations and Tables 1-3, as well as produce 15 pdf or eps files with the 15 figures.

typefig=1: produce PDF files
typefig=2: produce EPS files

Time needed: a few seconds

This analysis makes use of mortality data from the Human Mortality Database (HMD), Max Planck Institute for Demographic Research (Germany), University of California, Berkeley (USA), and French Institute for Demographic Studies (France). Available online at: www.mortality.org (last access May 14, 2024).

HMD data are licensed under a Creative Commons Attribution 4.0 International License. The data constructed by the HMD team are published under the CC BY 4.0 license.

All data used from HMD have been reorganized into an ASCII file "mortality.data.txt", with the number of deaths, population size and remaining life expectancy at ages 0-110 for both genders (men "M" and women "F") of 30 countries in 2019 and 2020.

Numbers of deaths have been rounded to the nearest integer higher or equal to 1.

Population size in 2019 was taken as the average population size on the 1st of January 2019 and 2020 and rounded to the nearest integer higher or equal to 1 and to the corresponding number of deaths.

Population size in 2020 was taken as the average population size on the 1st of January 2020 and 2021 and rounded to the nearest integer higher or equal to 1 and to the corresponding number of deaths.

Remaining life expectancies have been rounded to one decimal place.

The file "mortality.data.txt" is provided for the sake of reproducibility of results. To reanalyze such mortality data in future studies, it is however strongly recommended to download them directly from the HMD website, as HMD data are regularly updated.

One can find the following statement on the HMD website:

"Since the HMD data are updated on a regular basis (including corrections as needed), the preferable option is not to pass a copy of these data to other users. Instead, refer them to the HMD website, where they may download the data for themselves. This practice helps to prevent multiple outdated or incorrect versions. It also ensures that each user has full access to the information regarding the source and processing of mortality estimates, citation procedures, and any other useful documentation."

Our code has been tested on the following configuration:

```
R version 4.3.1 (2023-06-16)
Platform: aarch64-apple-darwin20 (64-bit)
Running under: macOS Ventura 13.5

Matrix products: default
BLAS:   /System/Library/Frameworks/Accelerate.framework/
Versions/A/Frameworks/vecLib.framework/Versions/A/
libBLAS.dylib
LAPACK: /Library/Frameworks/R.framework/Versions/4.3-arm64/
Resources/lib/libRlapack.dylib;  LAPACK version 3.11.0
```

```
locale:
[1] en_US.UTF-8/en_US.UTF-8/en_US.UTF-8/C/en_US.UTF-8/
en_US.UTF-8

time zone: Europe/Zurich
tzcode source: internal

attached base packages:
[1] stats     graphics  grDevices utils
[5] datasets  methods   base

loaded via a namespace (and not attached):
[1] compiler_4.3.1
```
