# Supplementary figures and images for: Years of Life Lost to COVID‐19 and Related Mortality Indicators: An Illustration in 30 Countries
Source: Biom J. 2024 Jul 13;66(5):e202300386. doi: 10.1002/bimj.202300386 (PMC12859533; doi:10.1002/bimj.202300386)

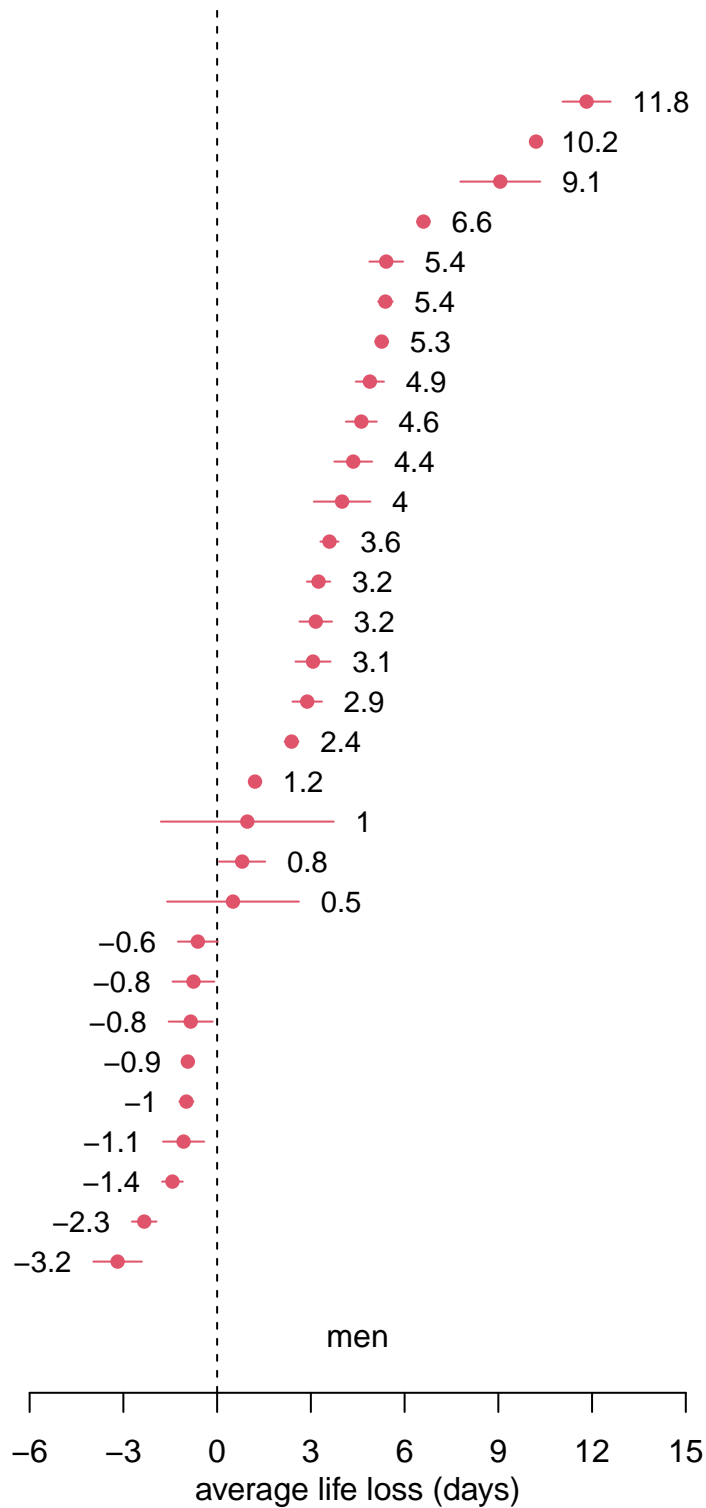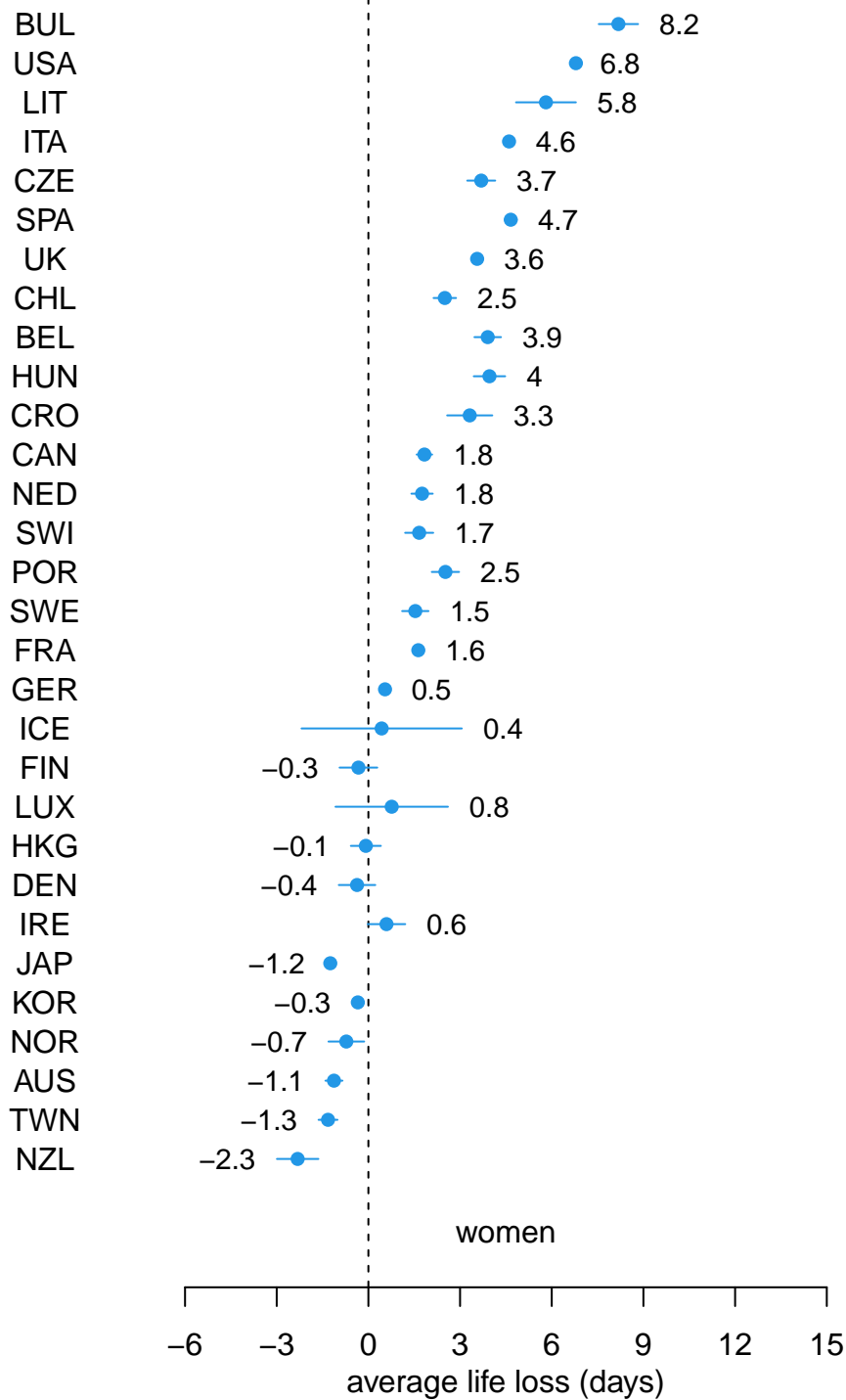

Supplement: Supplementary file 1 — Supporting Information [file BIMJ-66-e202300386-s002.zip › reproductibility/figure1.pdf]

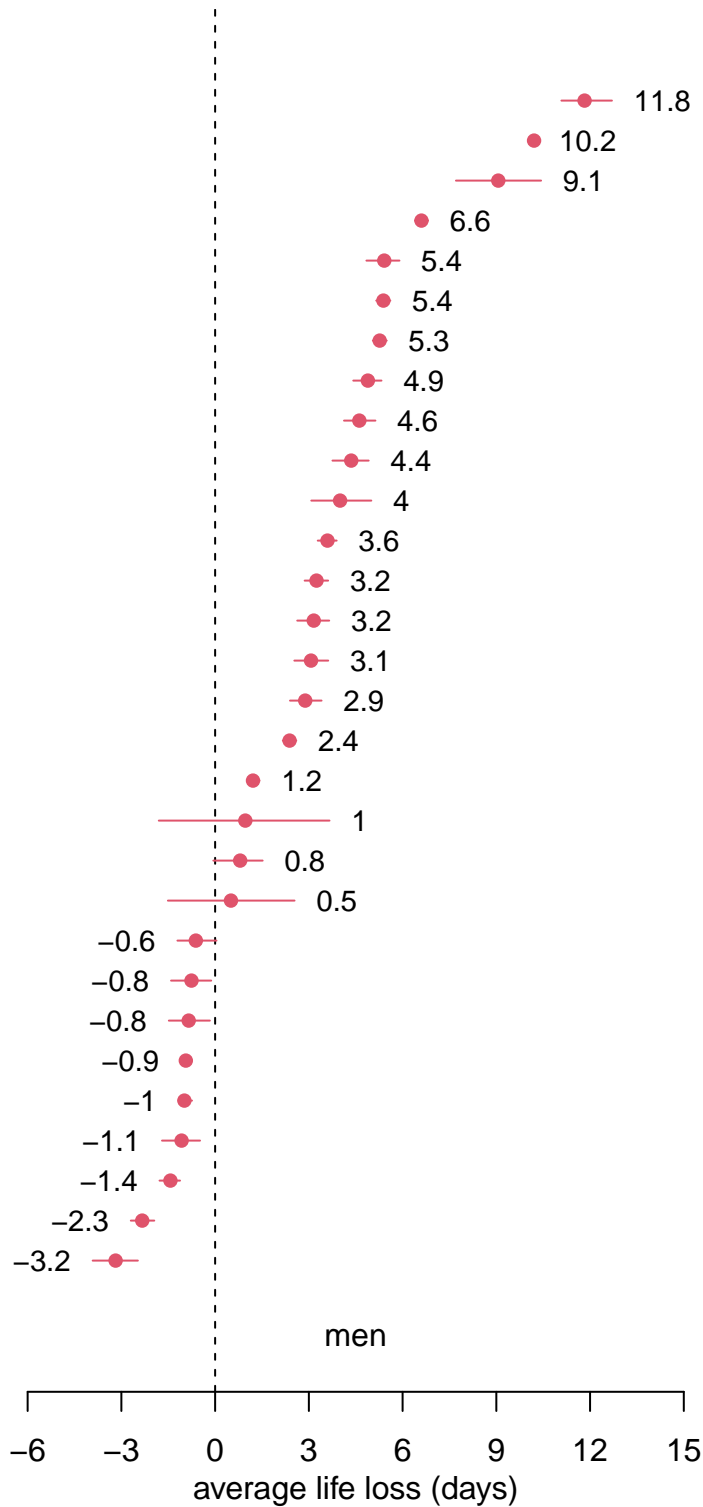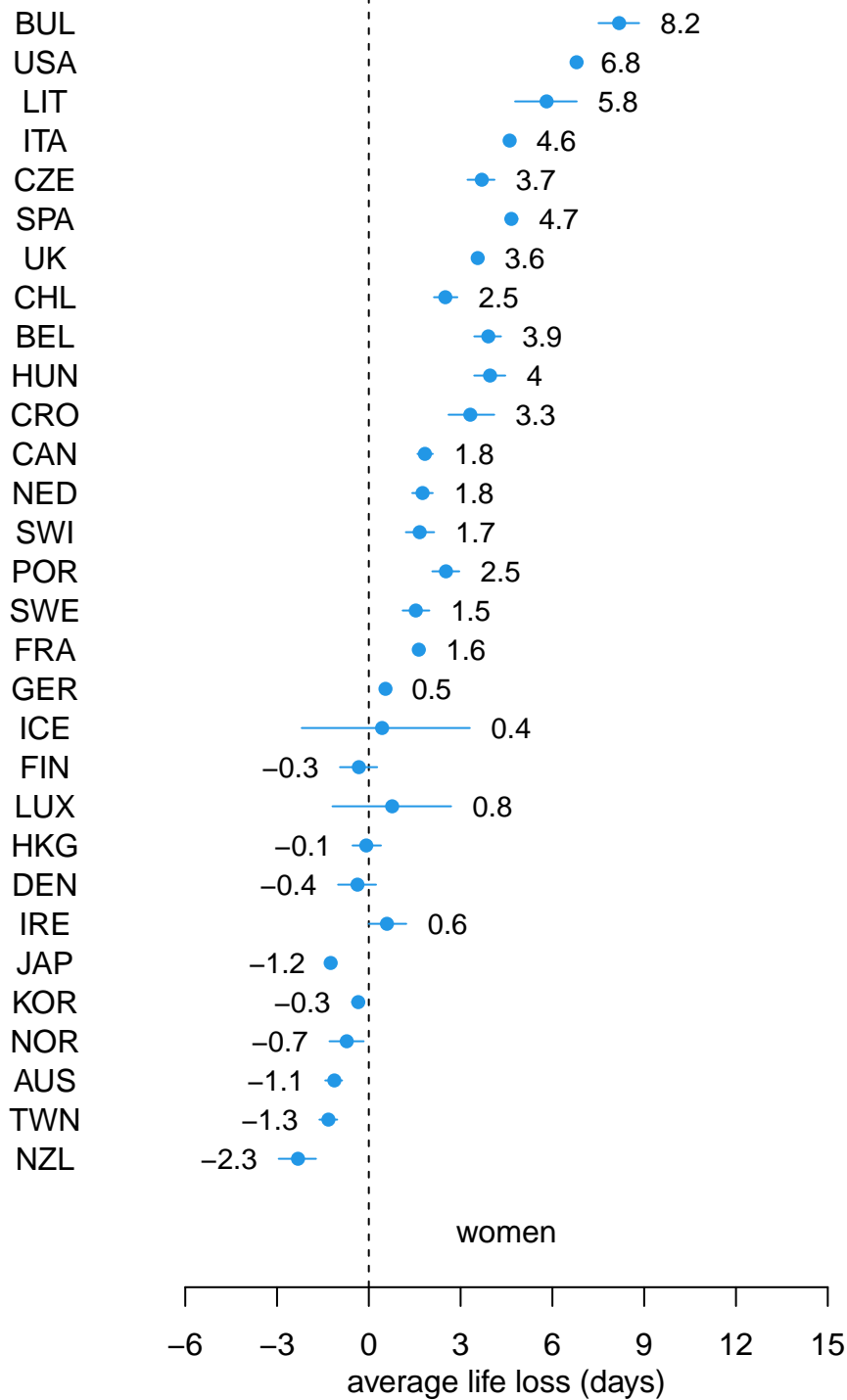

Supplement: Supplementary file 1 — Supporting Information [file BIMJ-66-e202300386-s002.zip › reproductibility/figure1s.pdf]

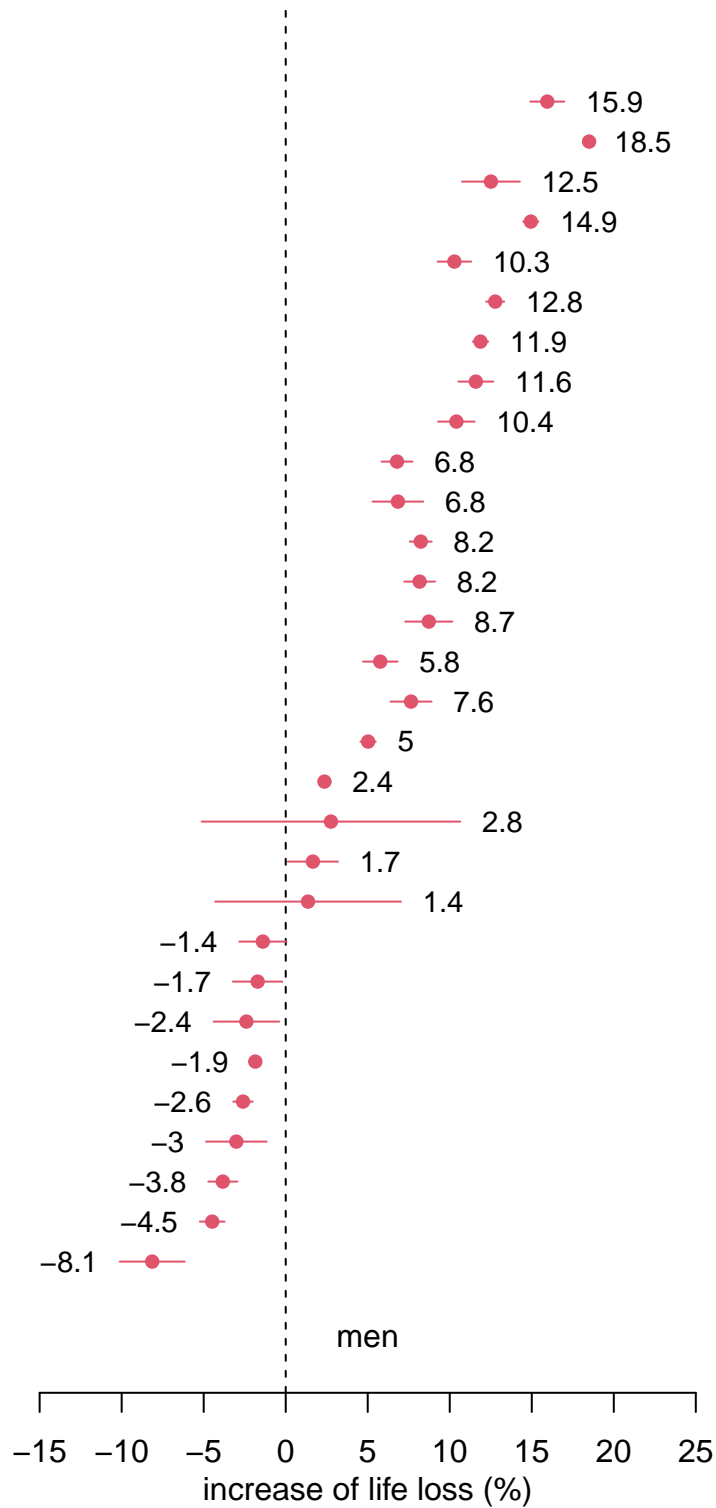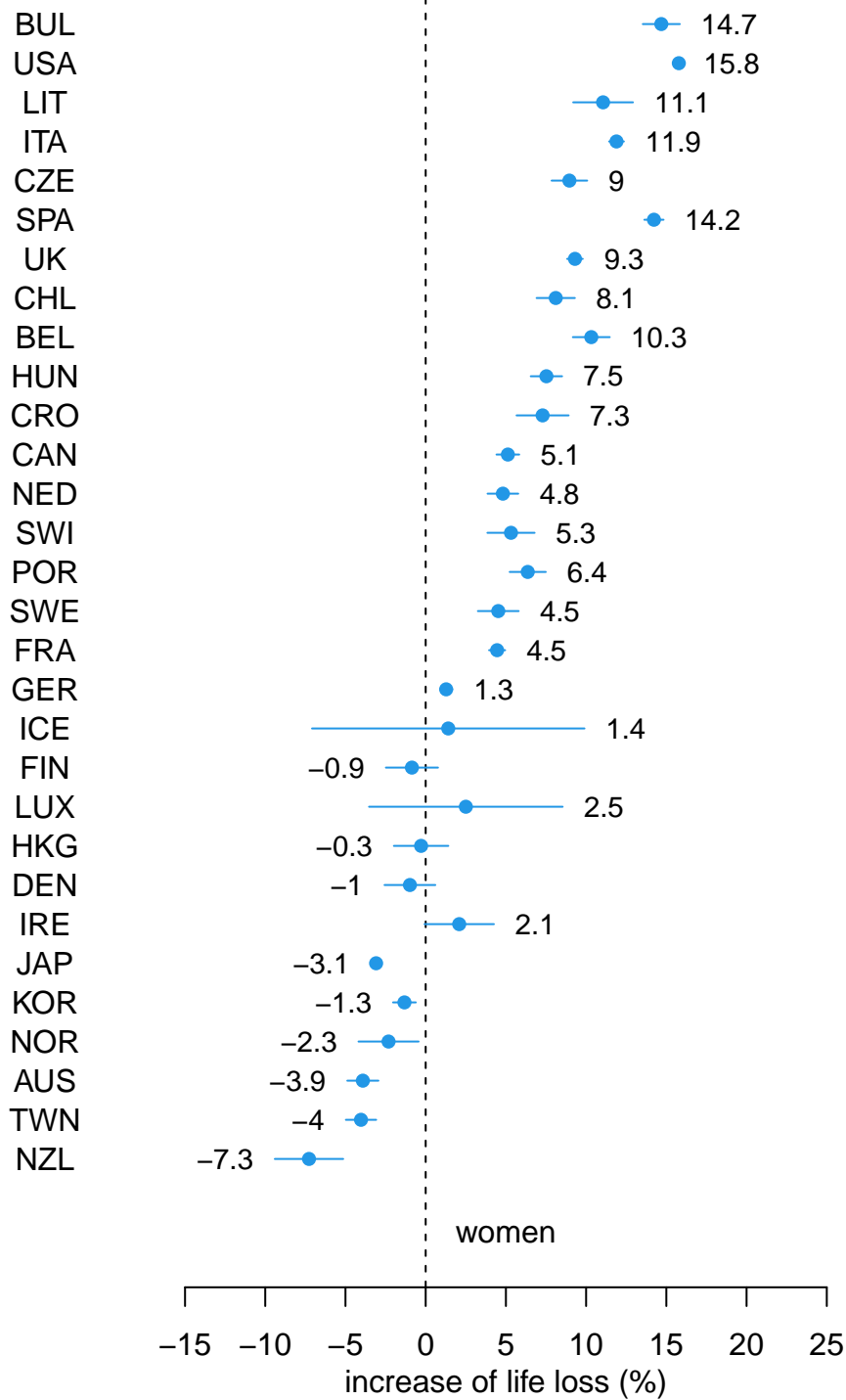

Supplement: Supplementary file 1 — Supporting Information [file BIMJ-66-e202300386-s002.zip › reproductibility/figure2.pdf]

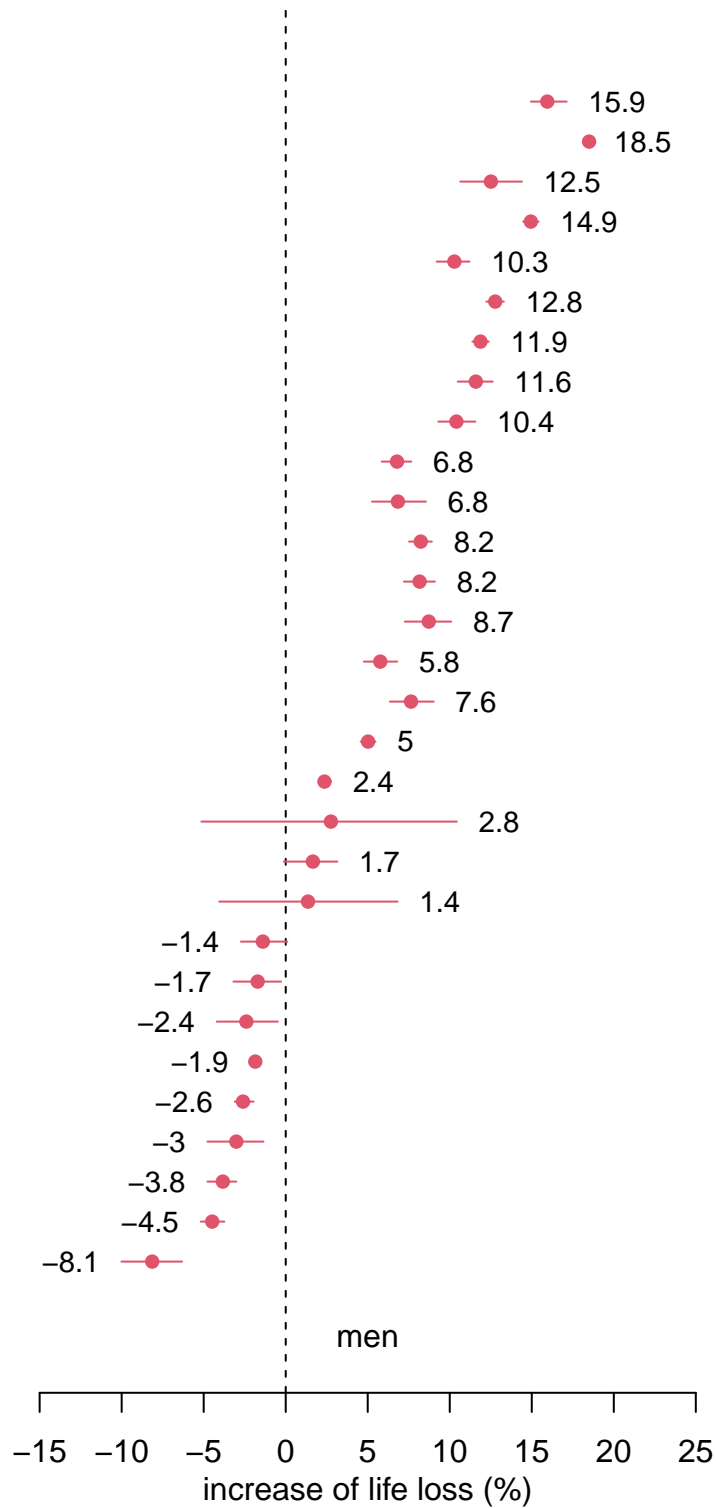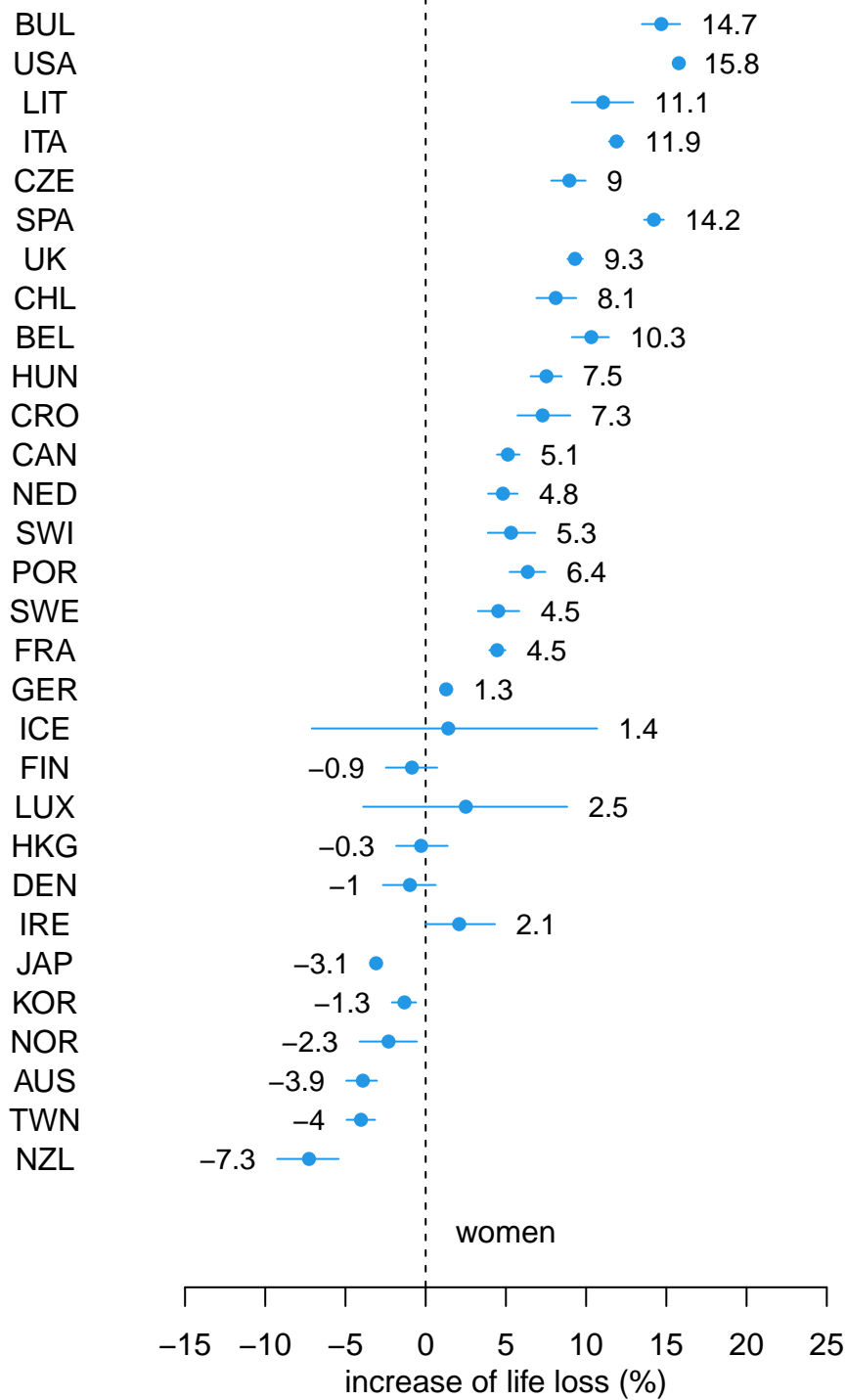

Supplement: Supplementary file 1 — Supporting Information [file BIMJ-66-e202300386-s002.zip › reproductibility/figure2s.pdf]

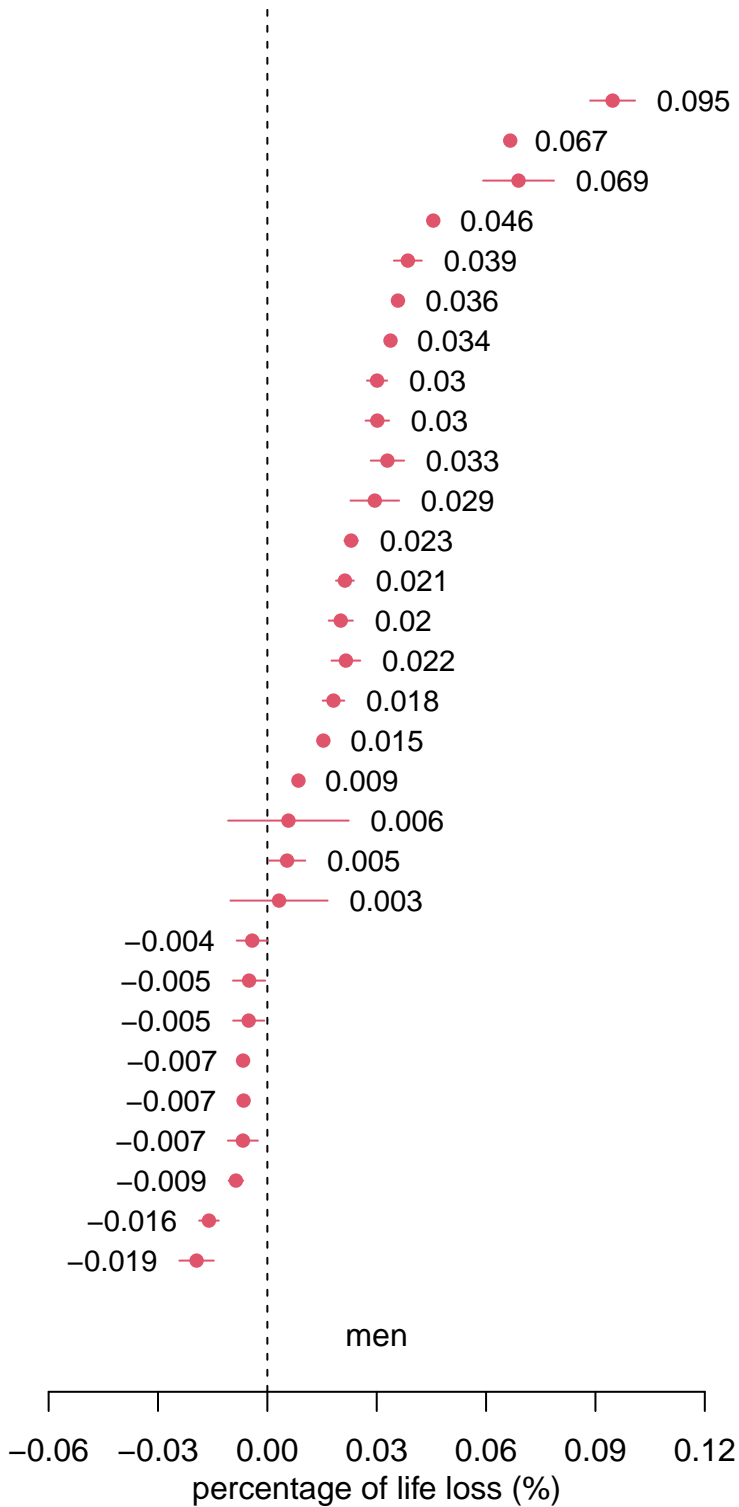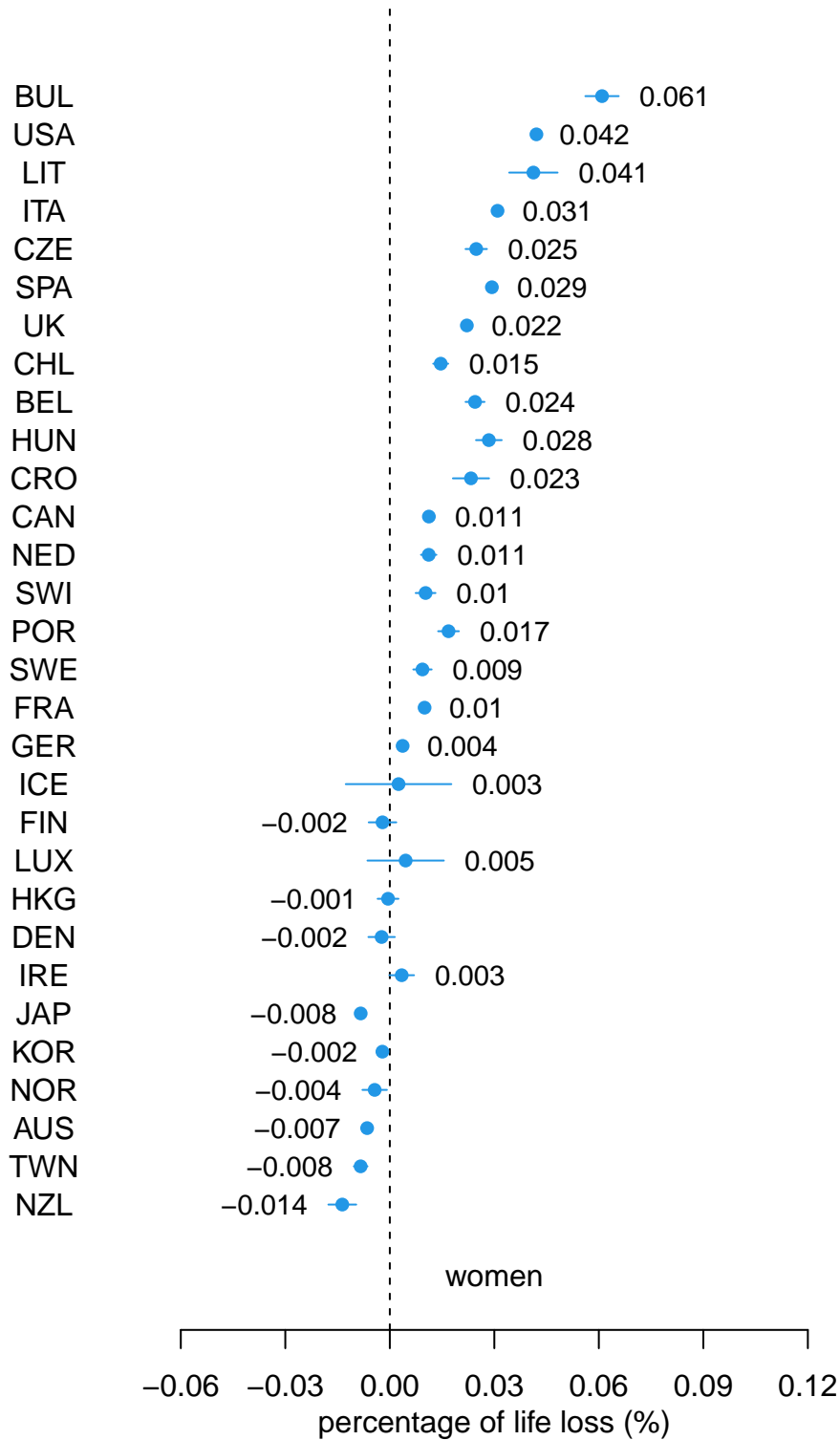

Supplement: Supplementary file 1 — Supporting Information [file BIMJ-66-e202300386-s002.zip › reproductibility/figure3.pdf]

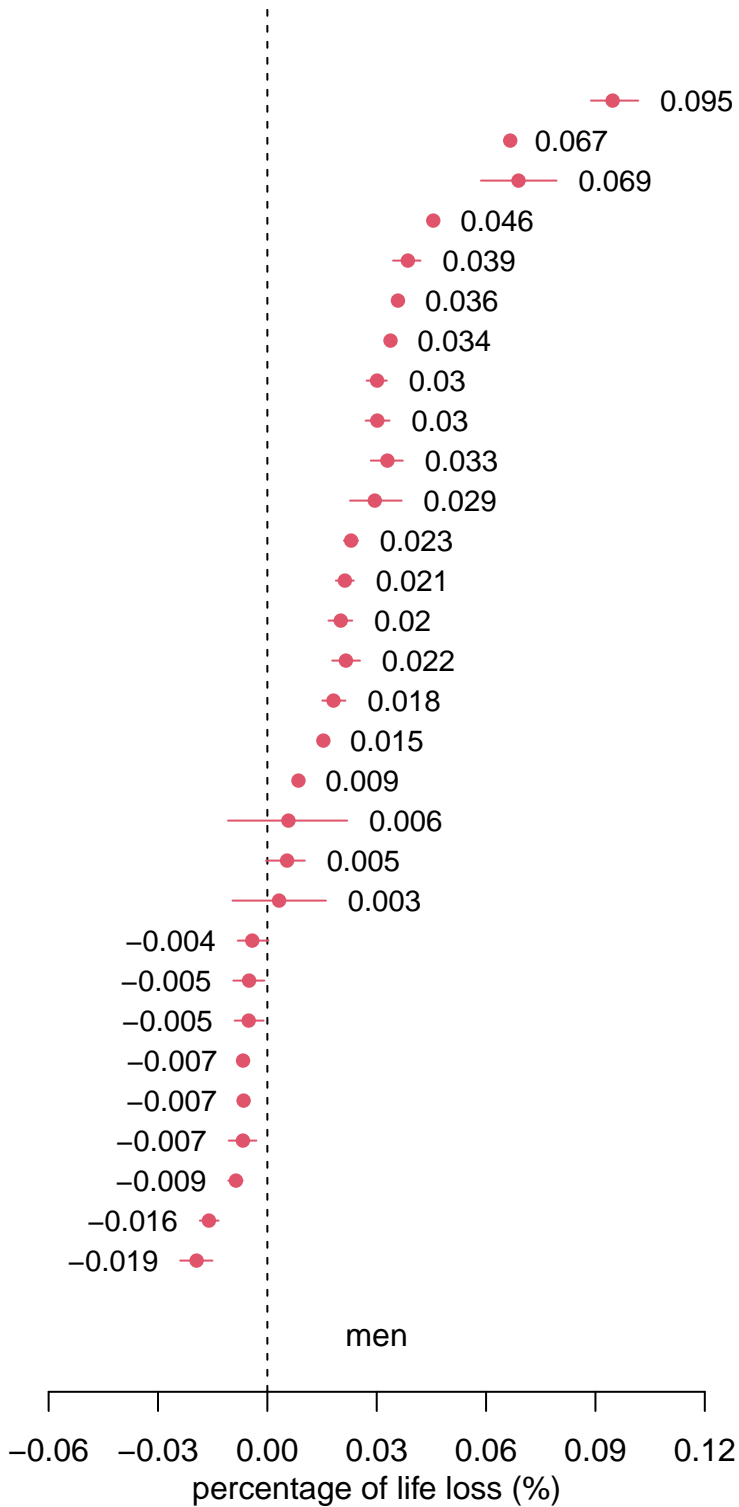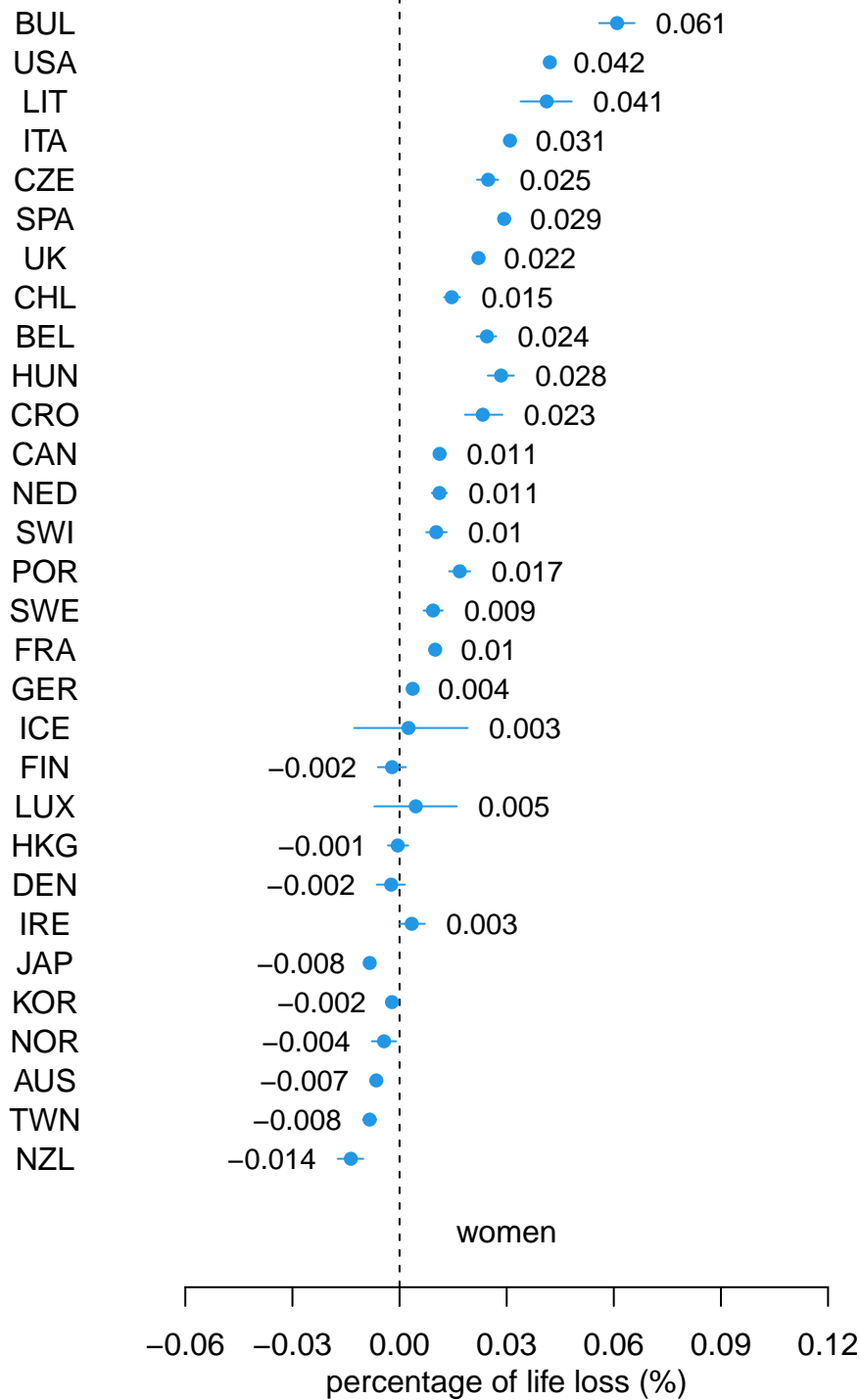

Supplement: Supplementary file 1 — Supporting Information [file BIMJ-66-e202300386-s002.zip › reproductibility/figure3s.pdf]

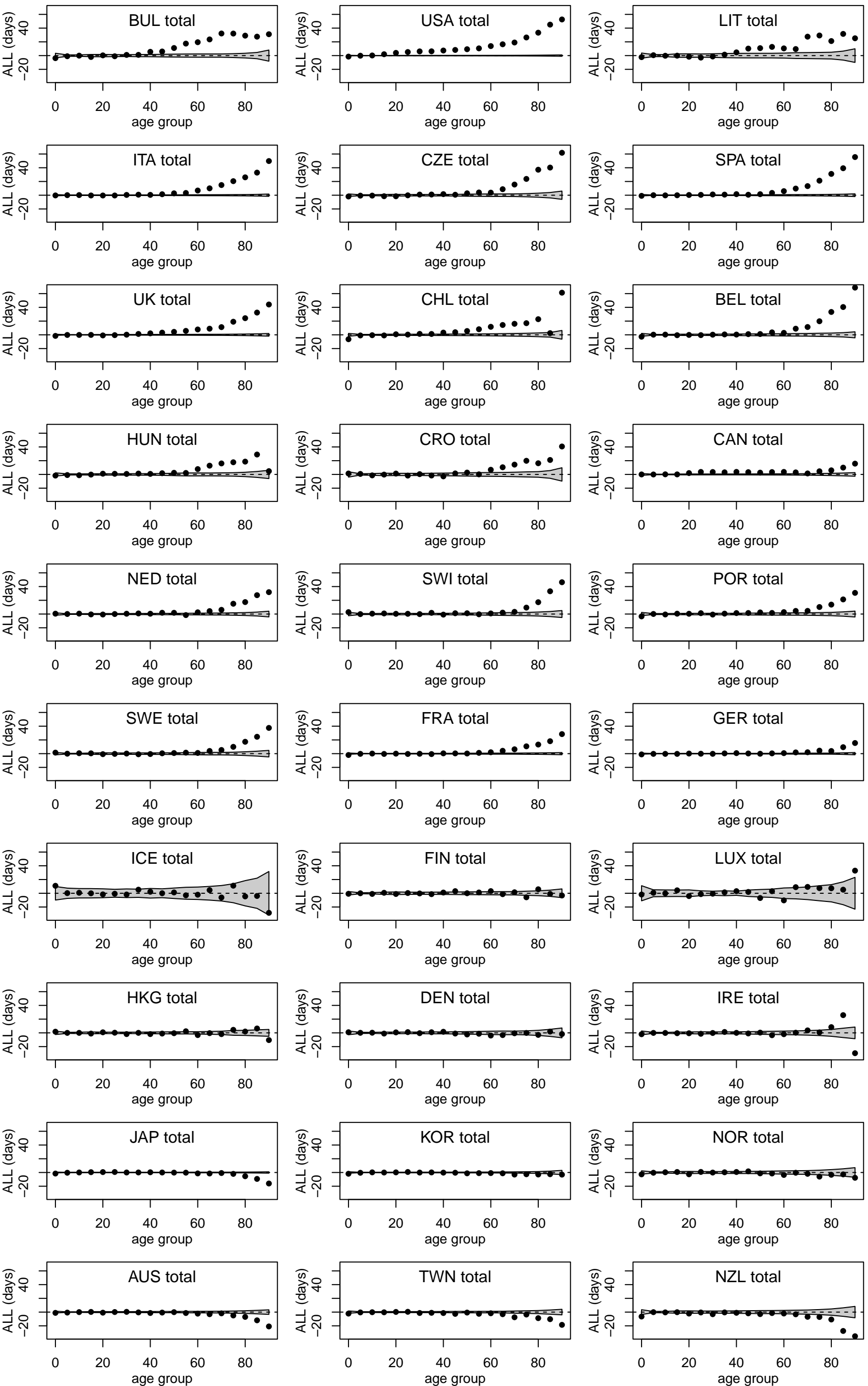

Supplement: Supplementary file 1 — Supporting Information [file BIMJ-66-e202300386-s002.zip › reproductibility/figure4.pdf]

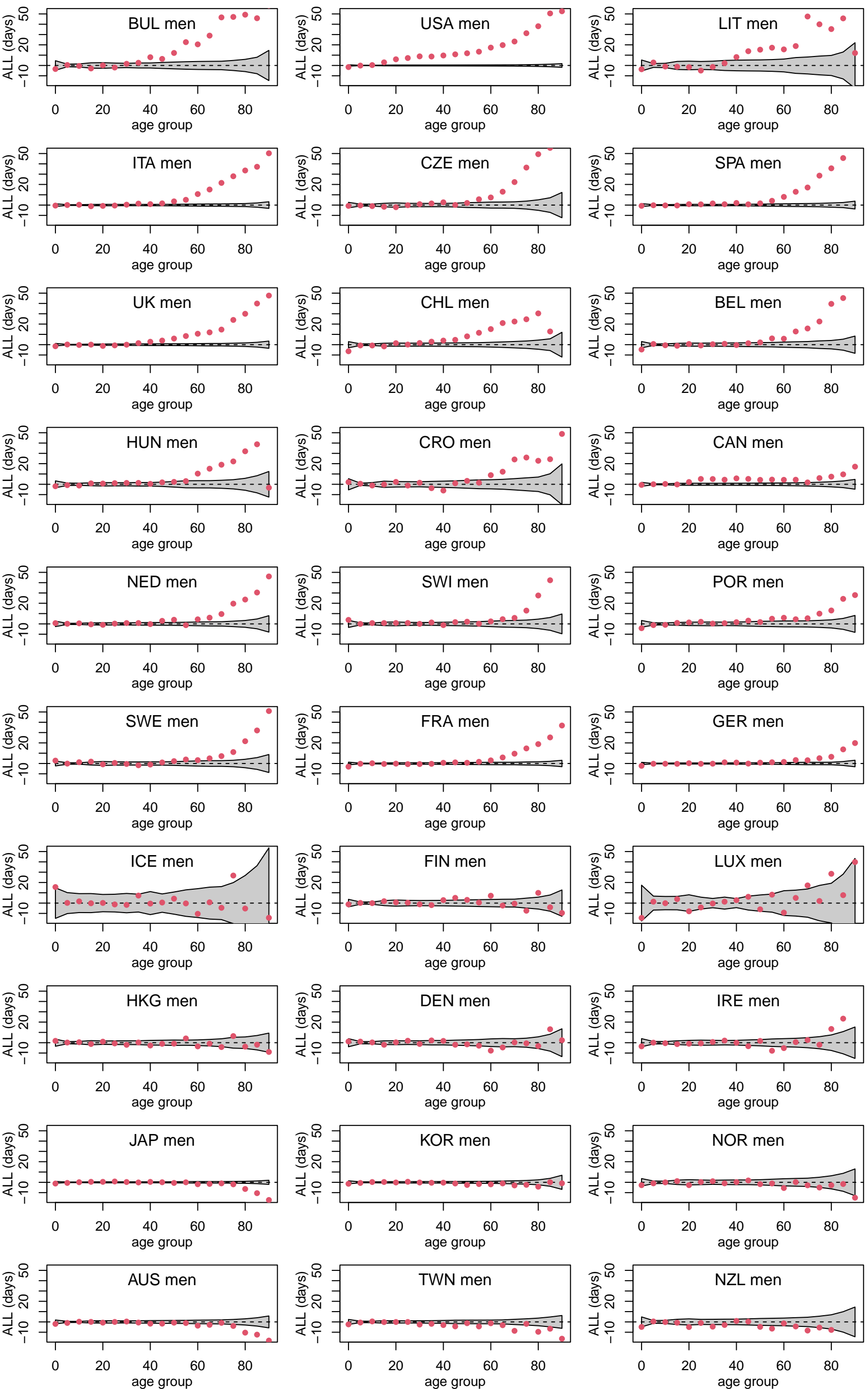

Supplement: Supplementary file 1 — Supporting Information [file BIMJ-66-e202300386-s002.zip › reproductibility/figure4s.pdf]

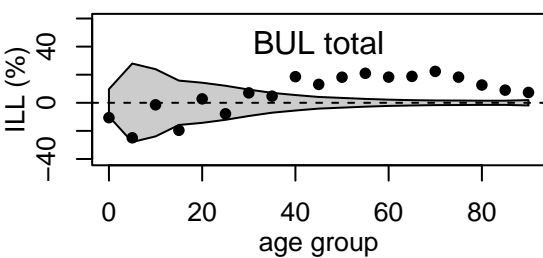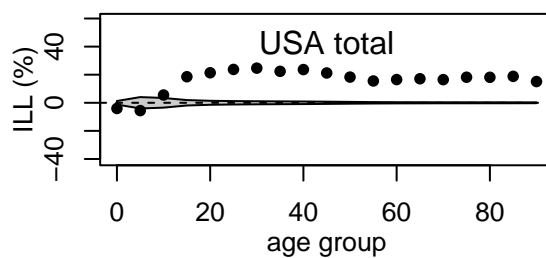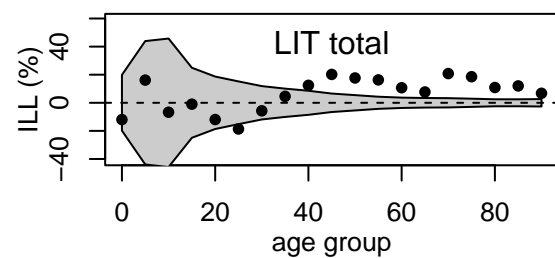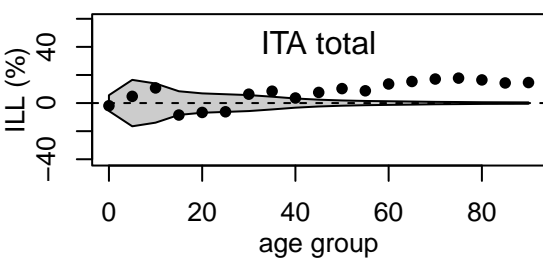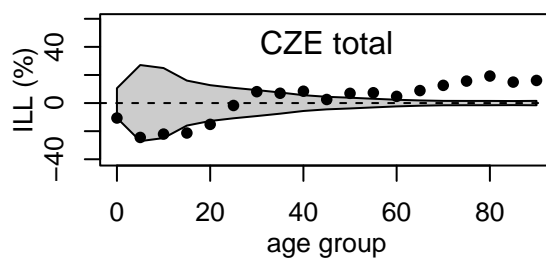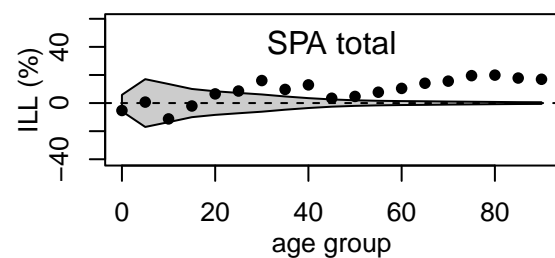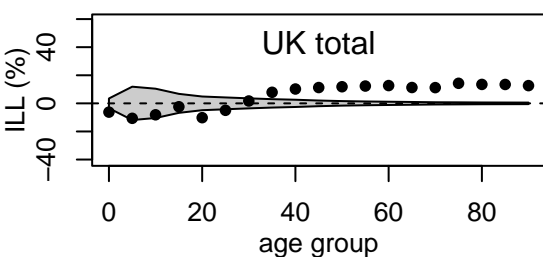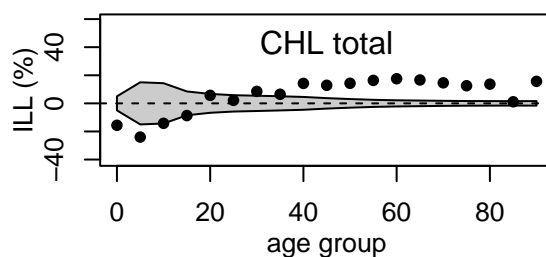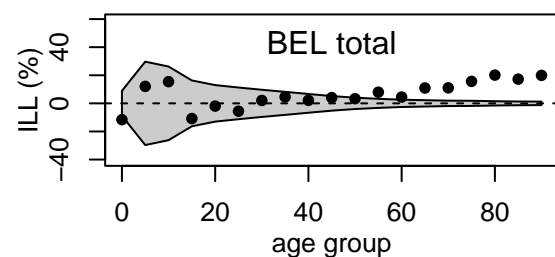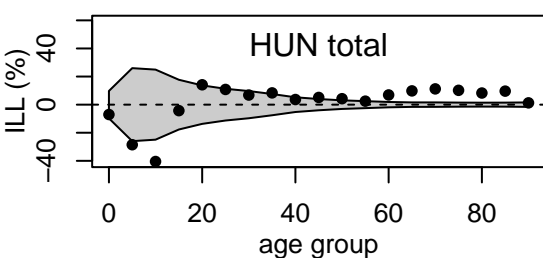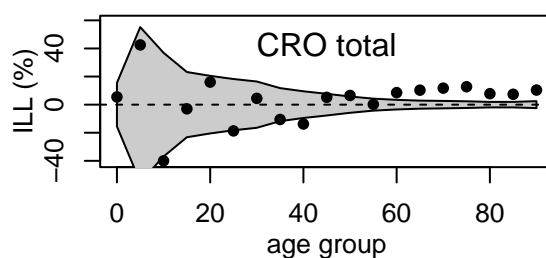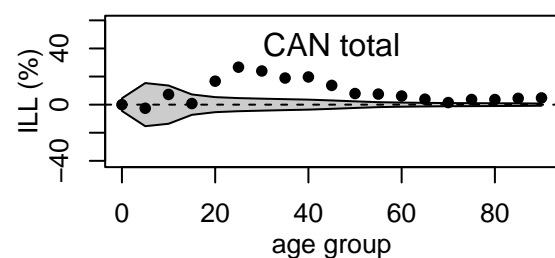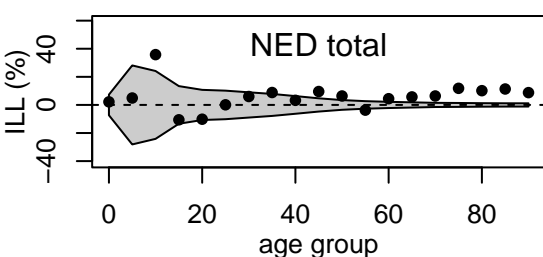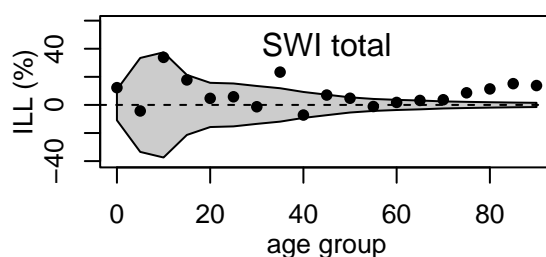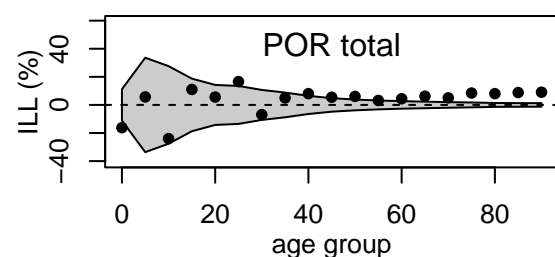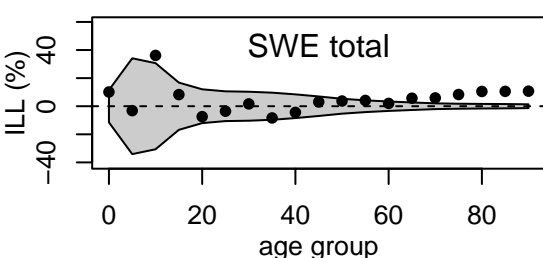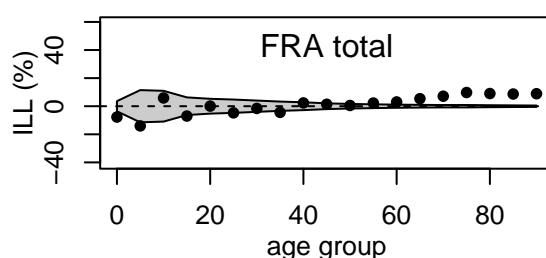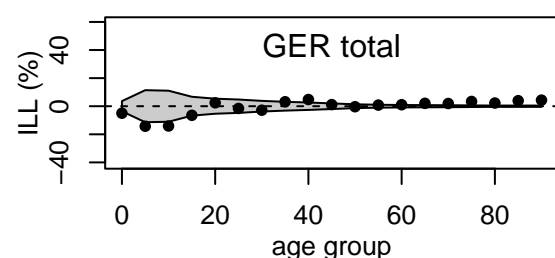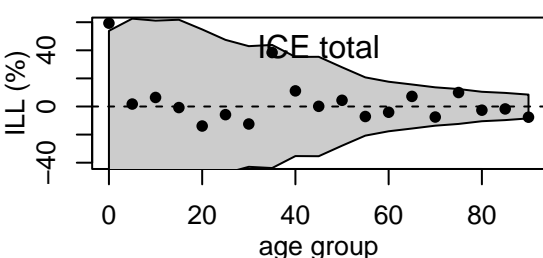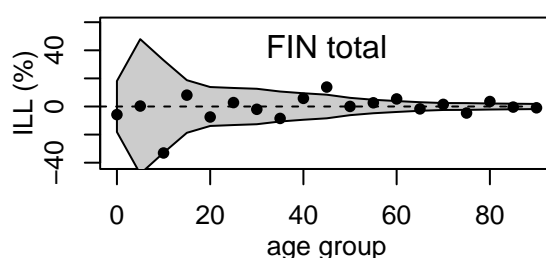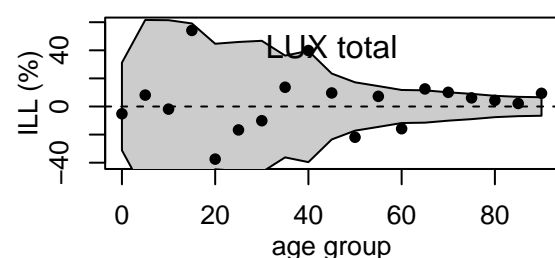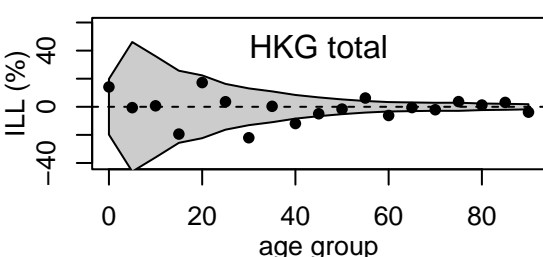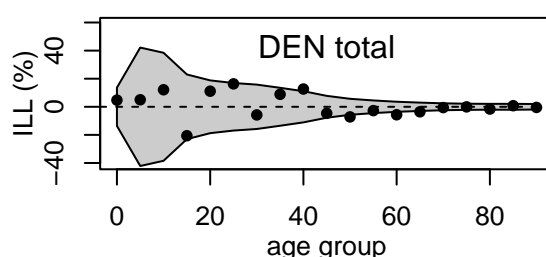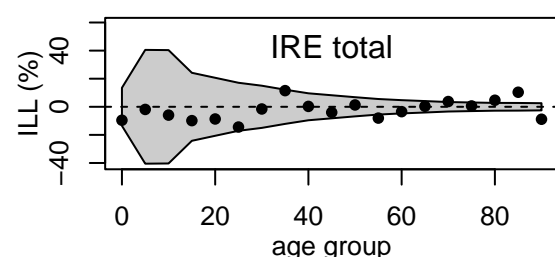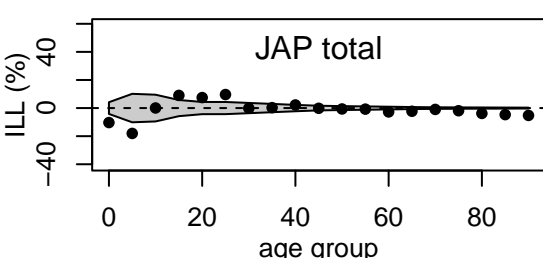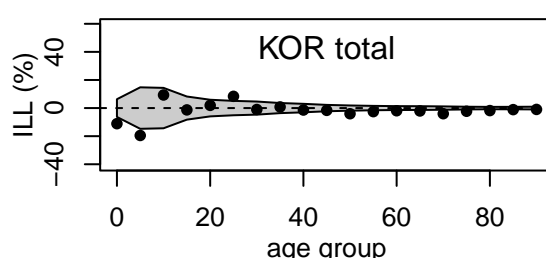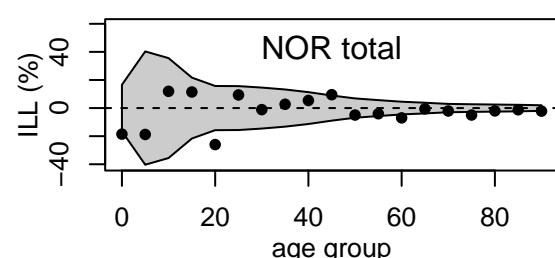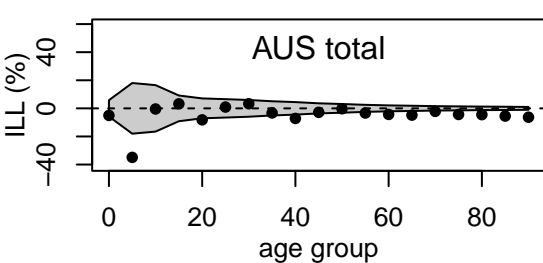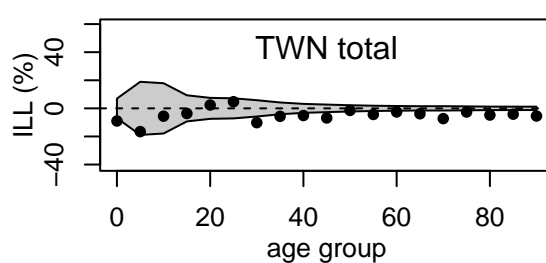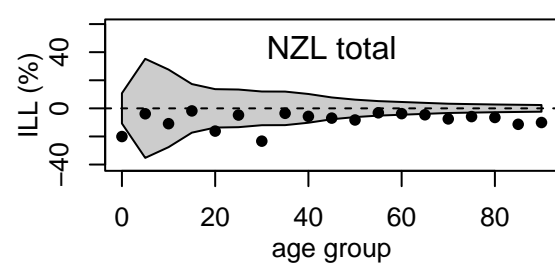

Supplement: Supplementary file 1 — Supporting Information [file BIMJ-66-e202300386-s002.zip › reproductibility/figure5.pdf]

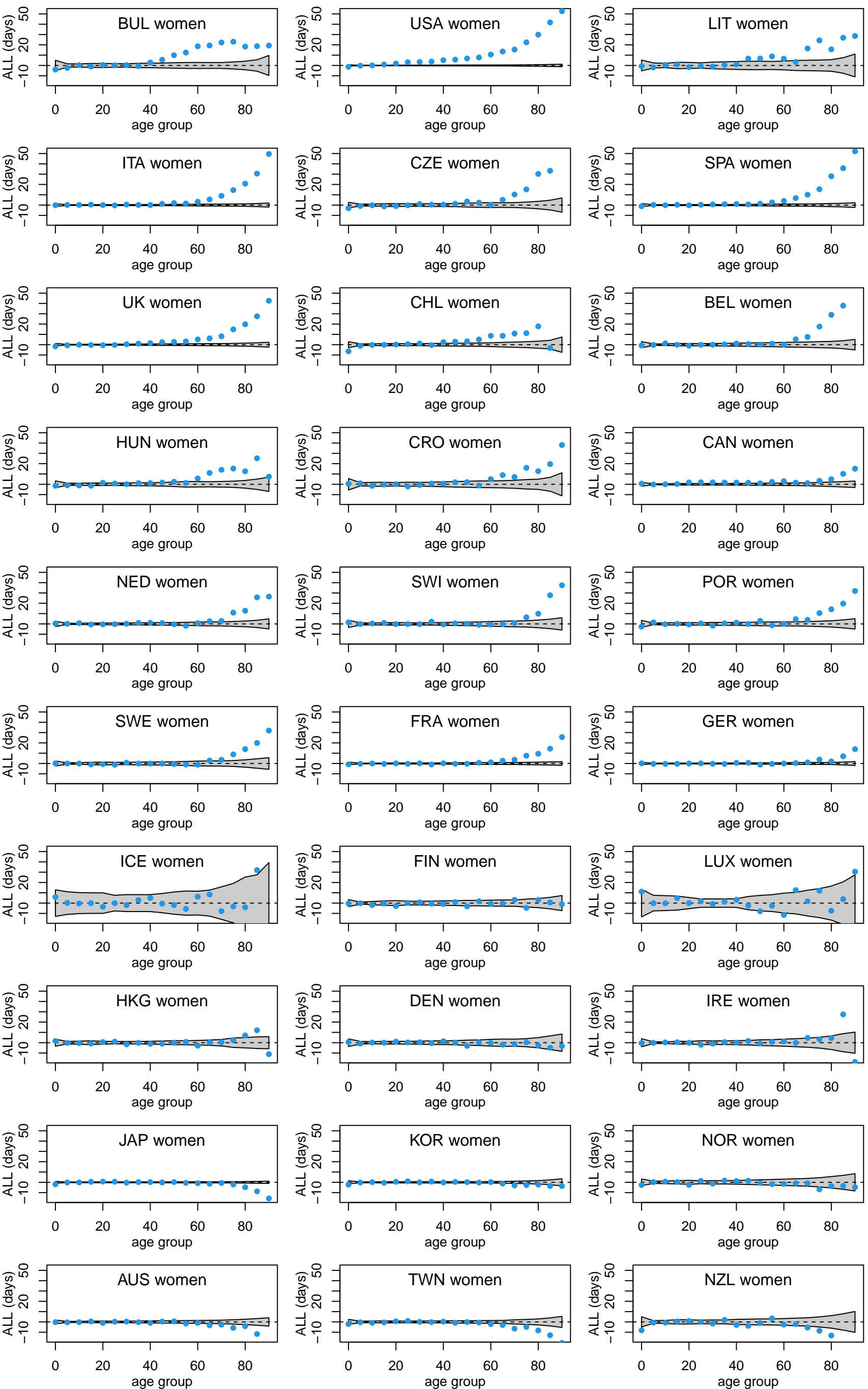

Supplement: Supplementary file 1 — Supporting Information [file BIMJ-66-e202300386-s002.zip › reproductibility/figure5s.pdf]

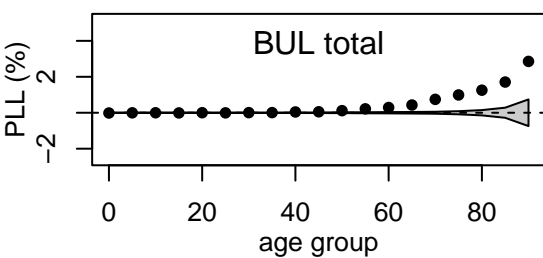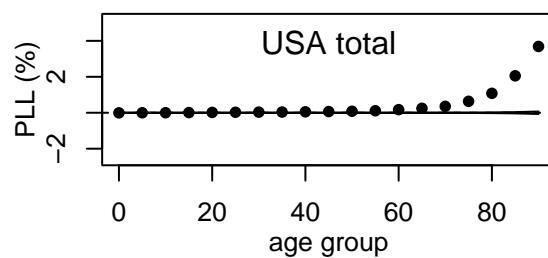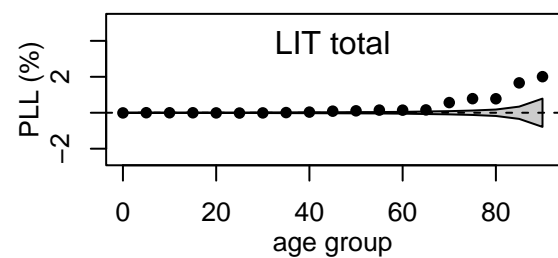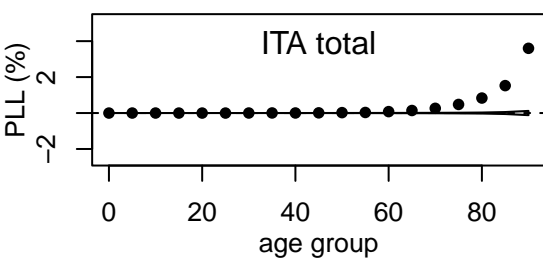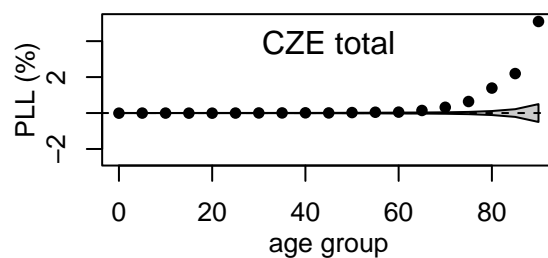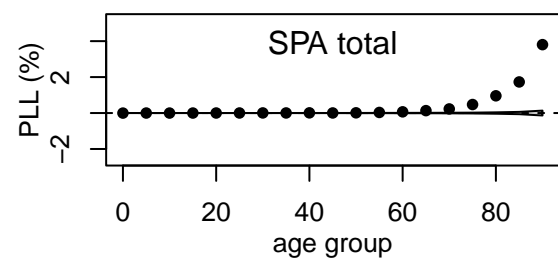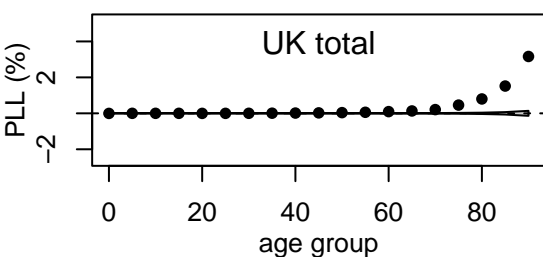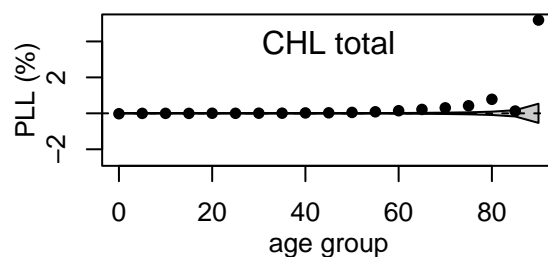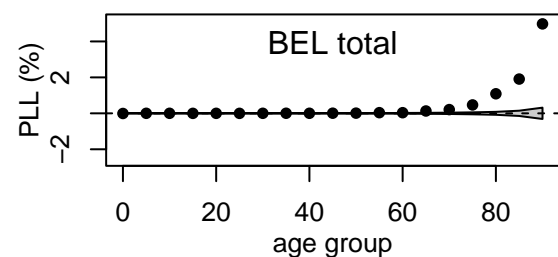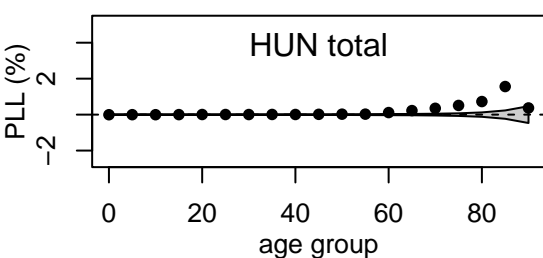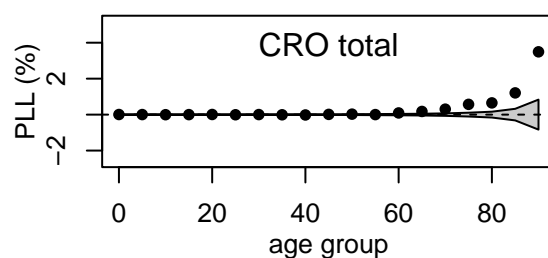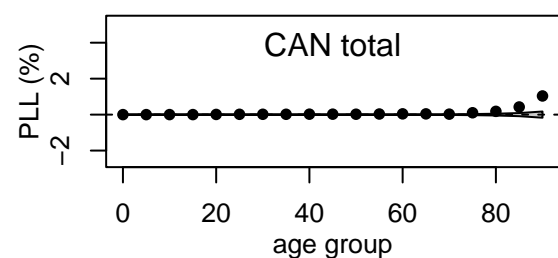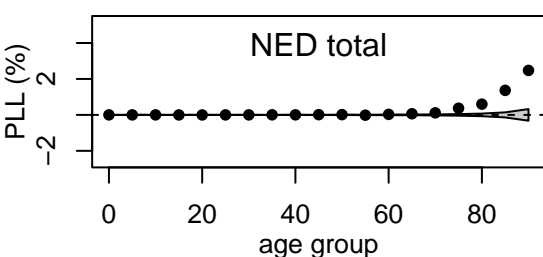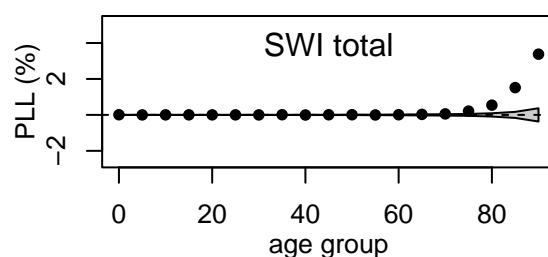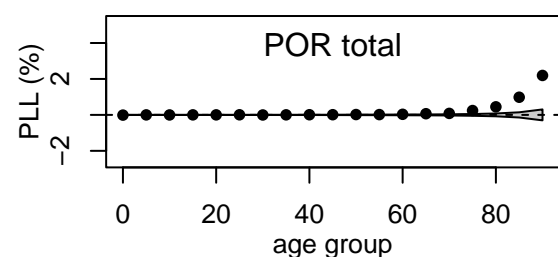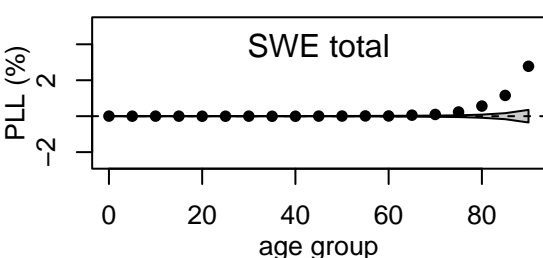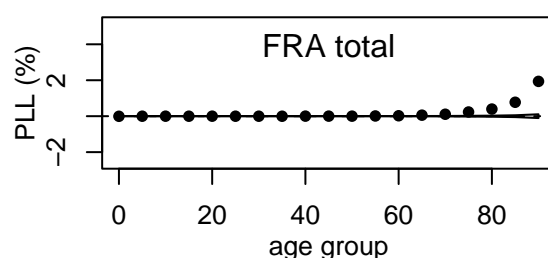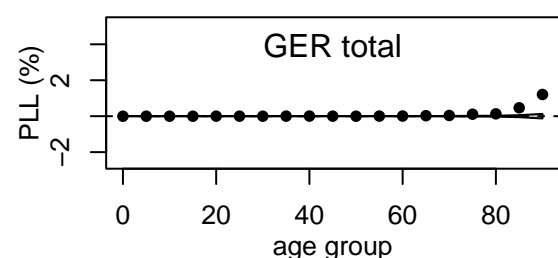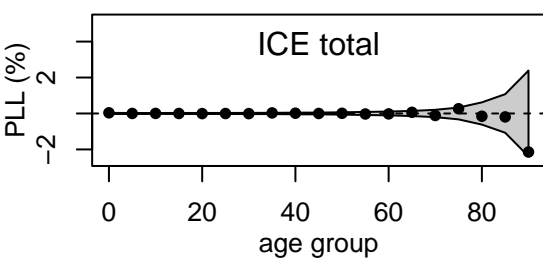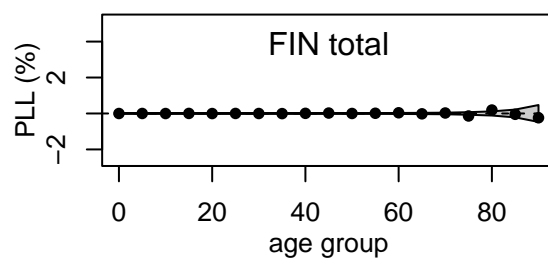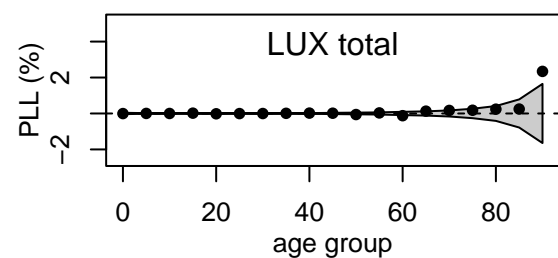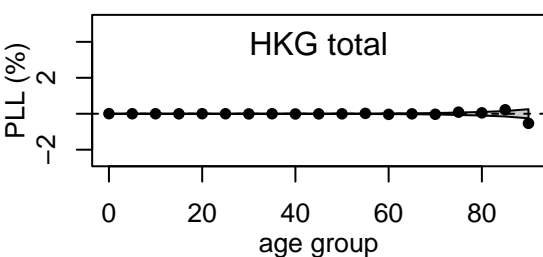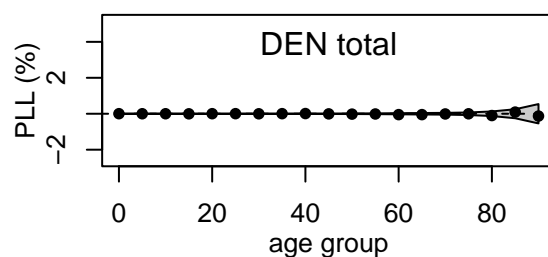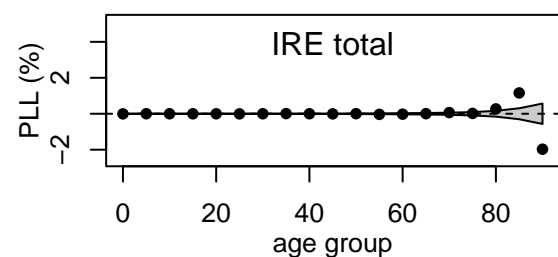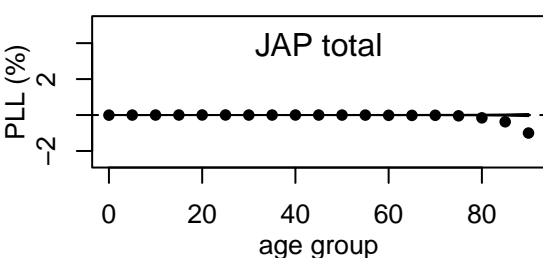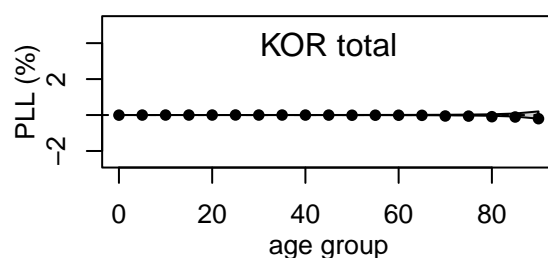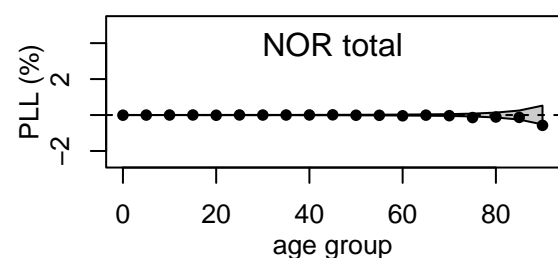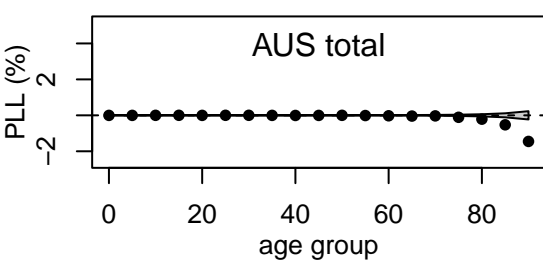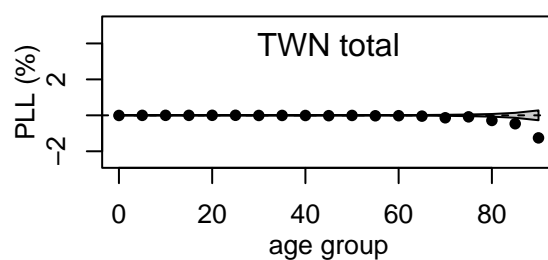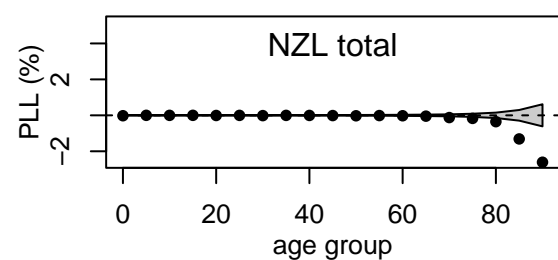

Supplement: Supplementary file 1 — Supporting Information [file BIMJ-66-e202300386-s002.zip › reproductibility/figure6.pdf]

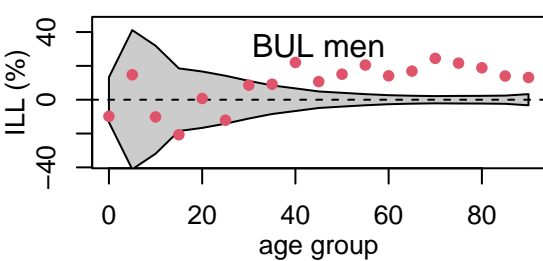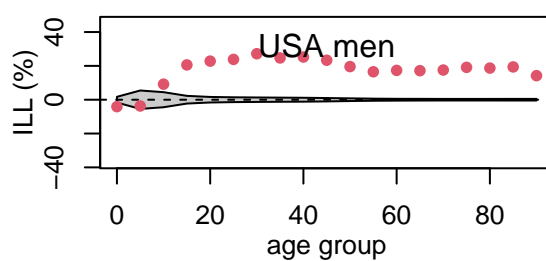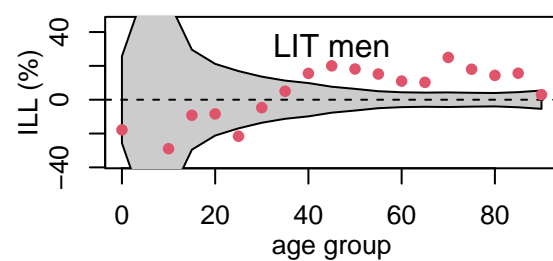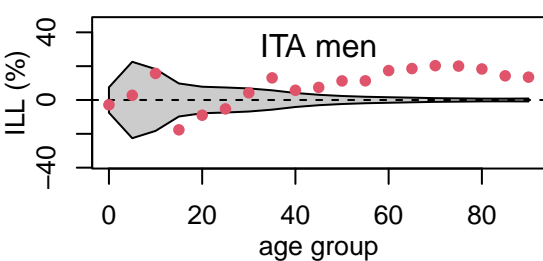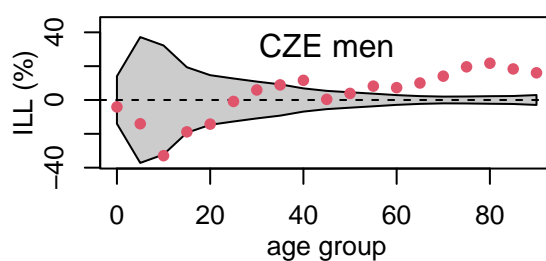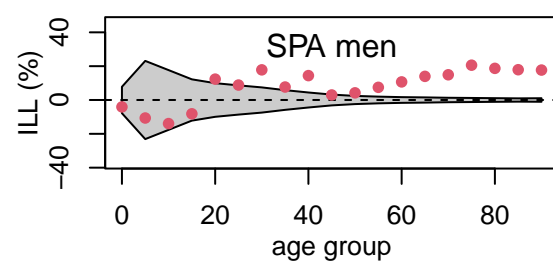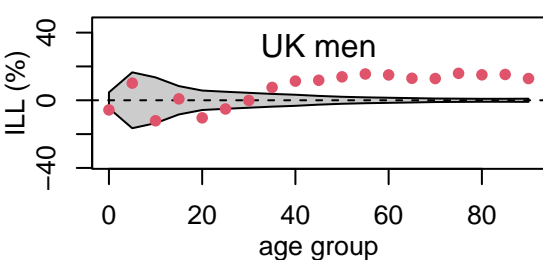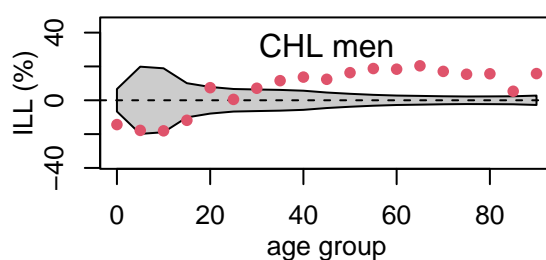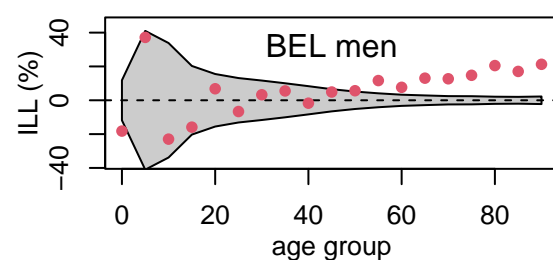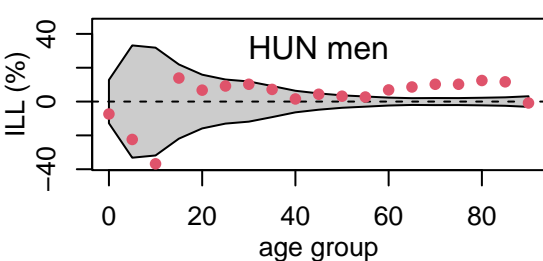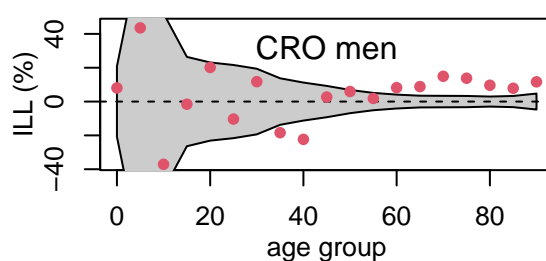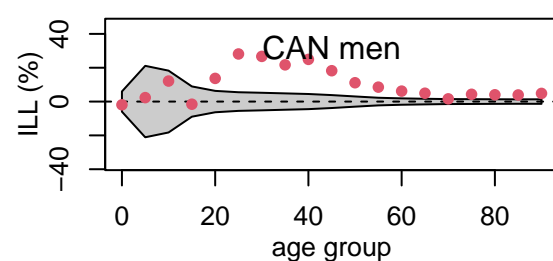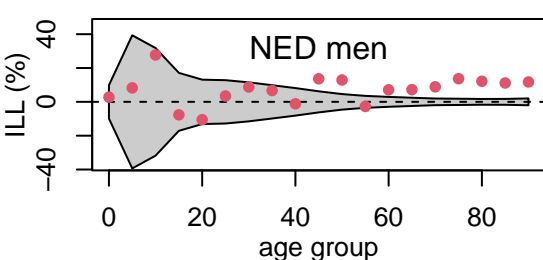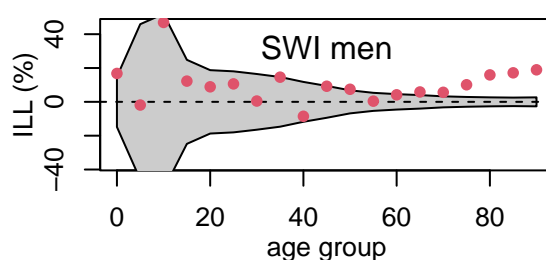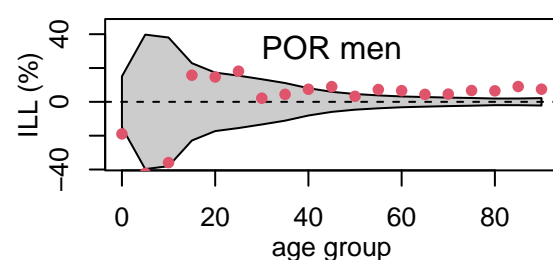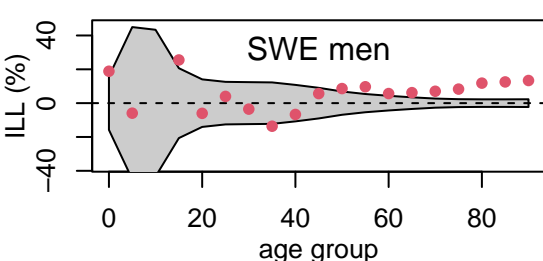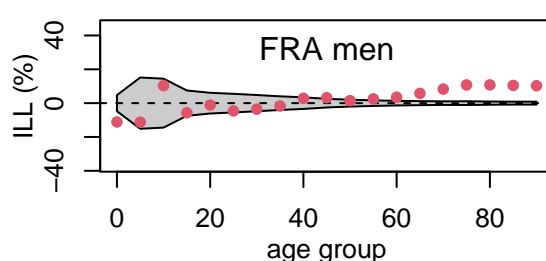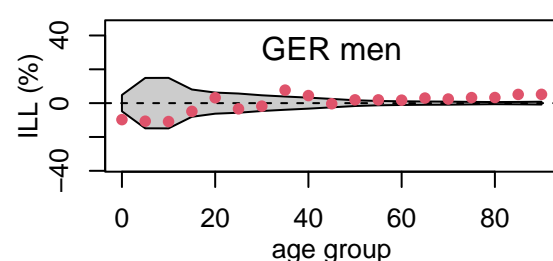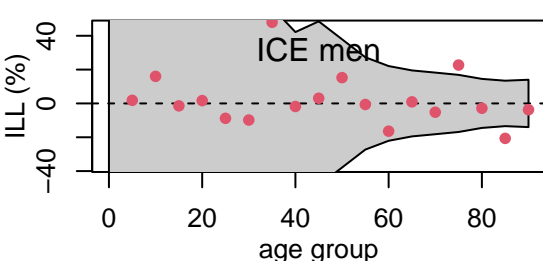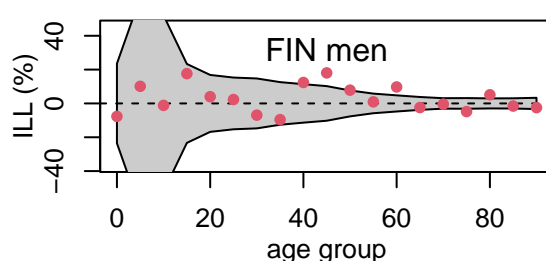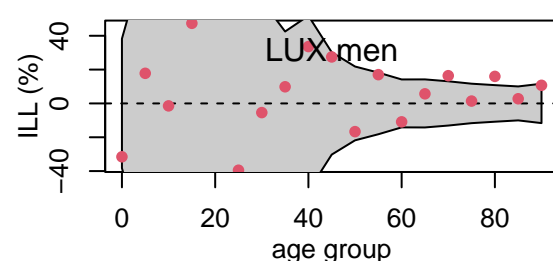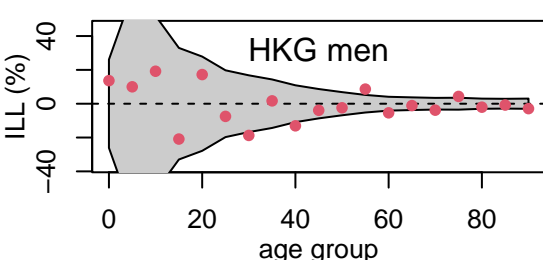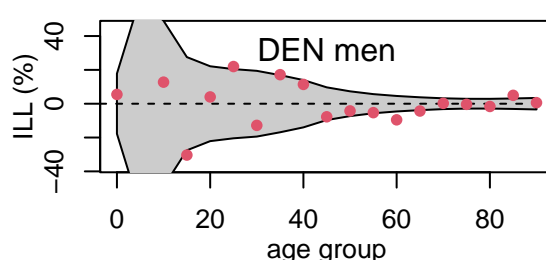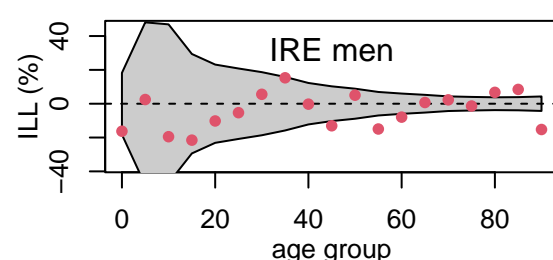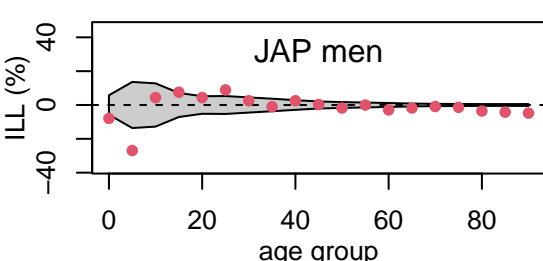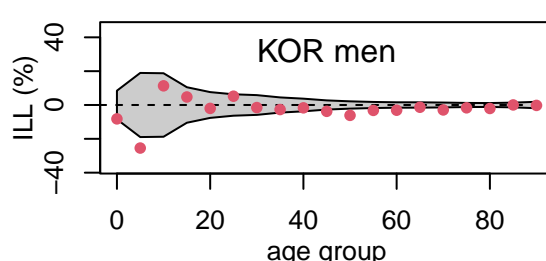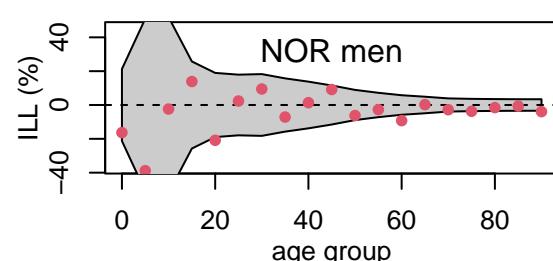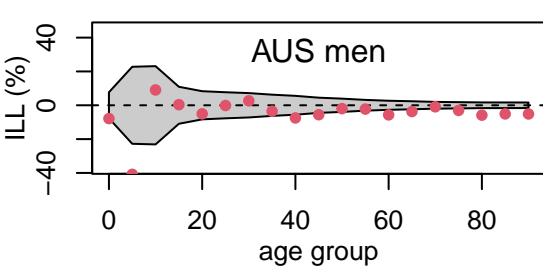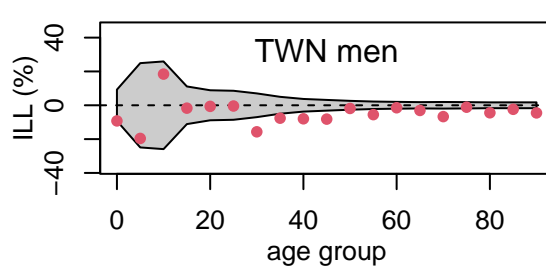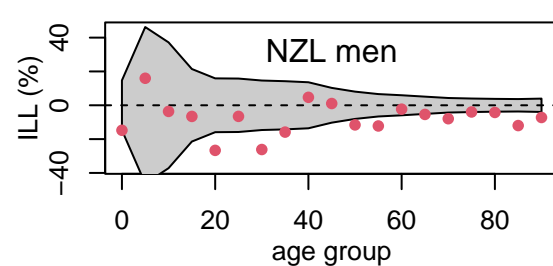

Supplement: Supplementary file 1 — Supporting Information [file BIMJ-66-e202300386-s002.zip › reproductibility/figure6s.pdf]

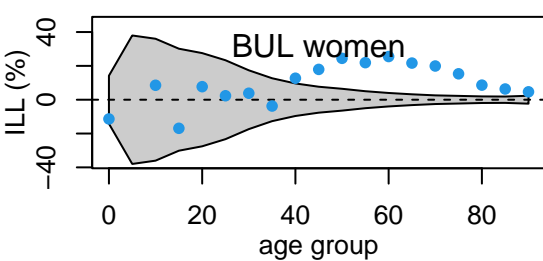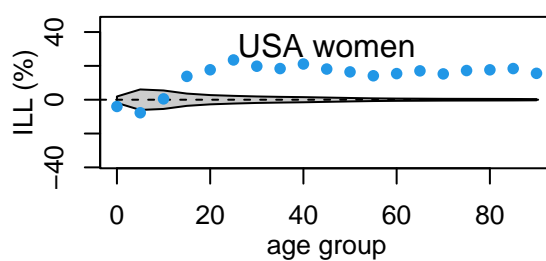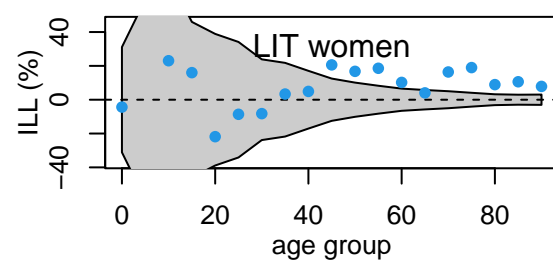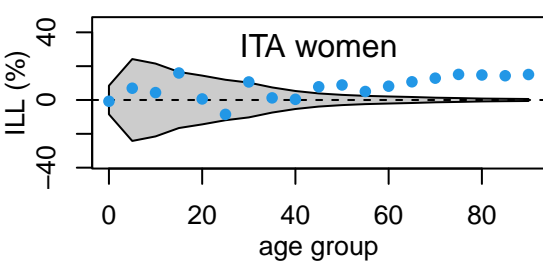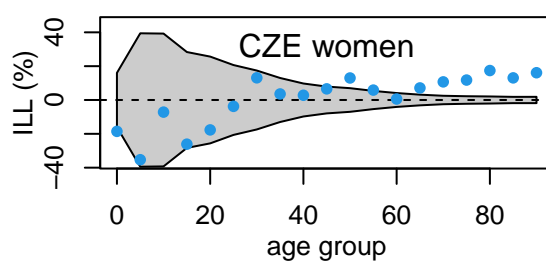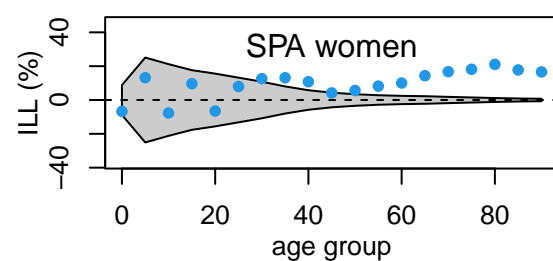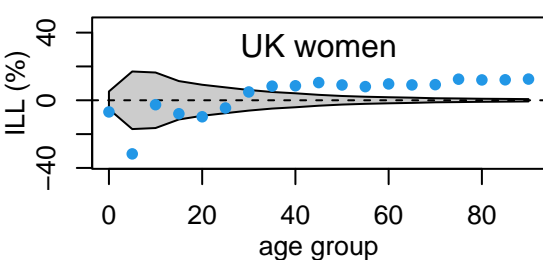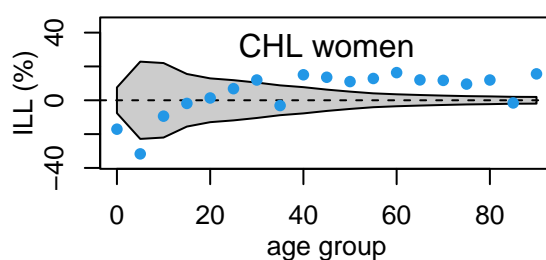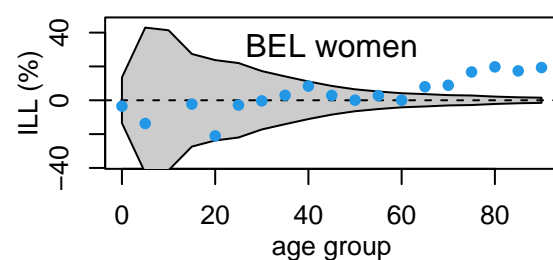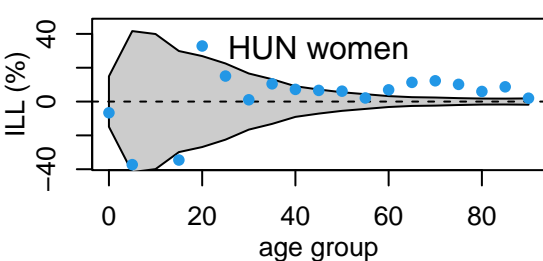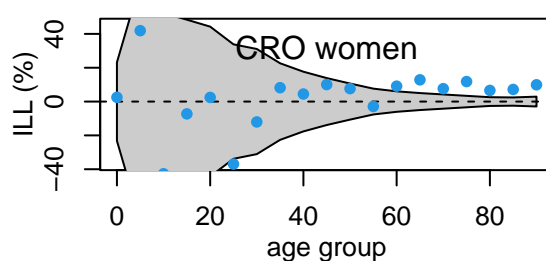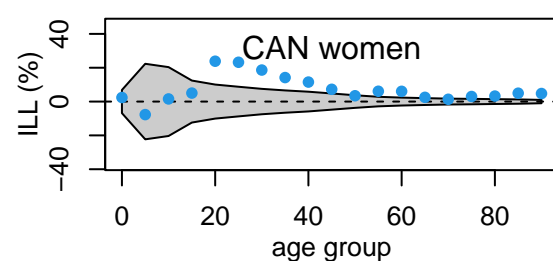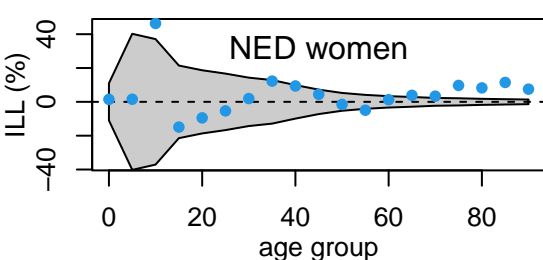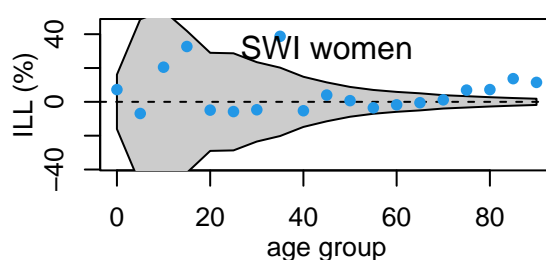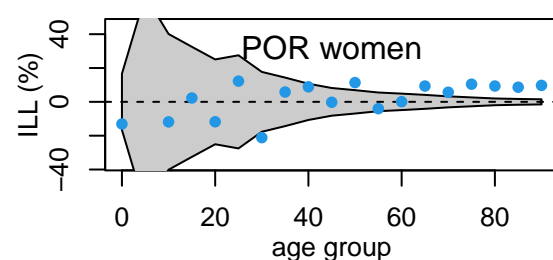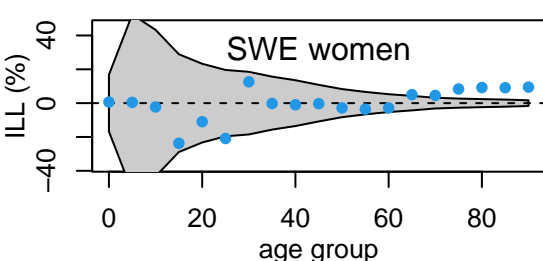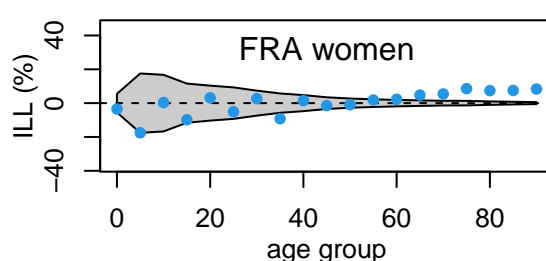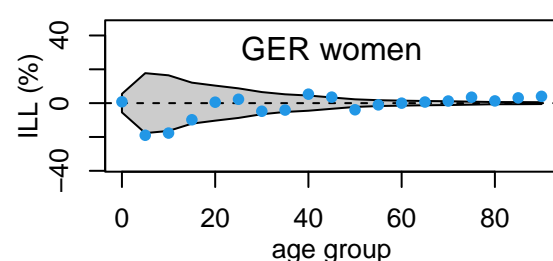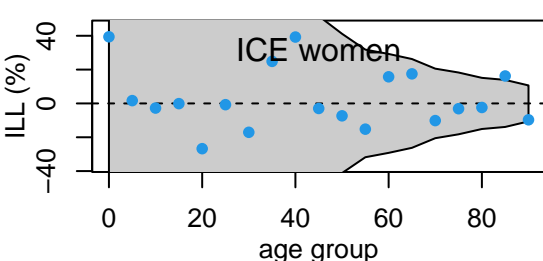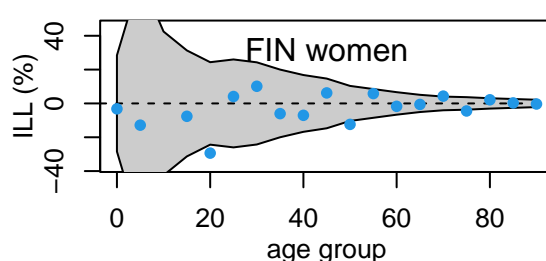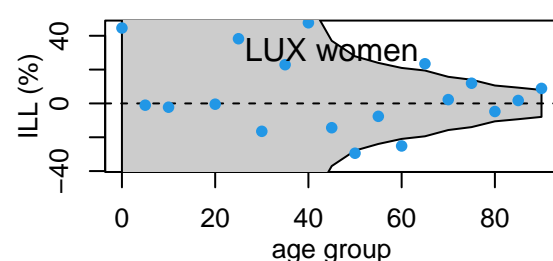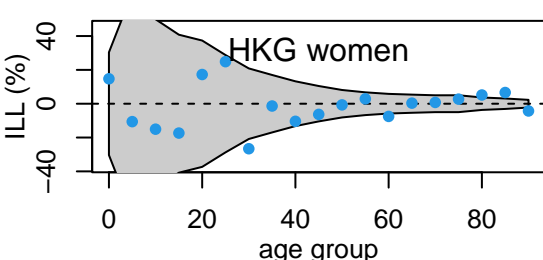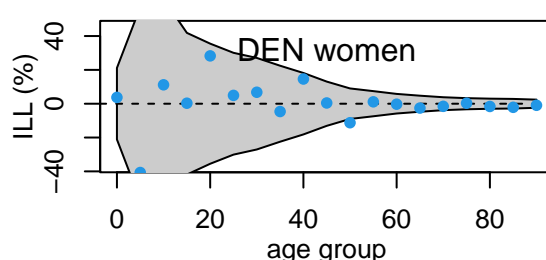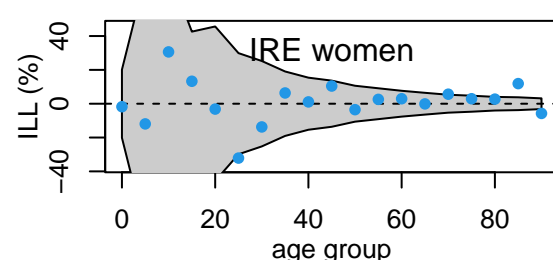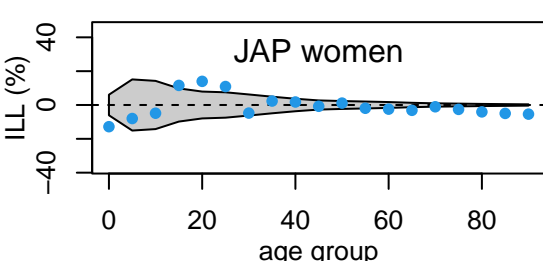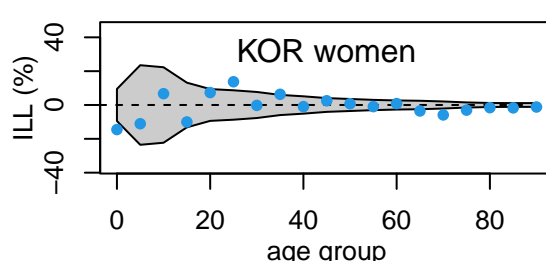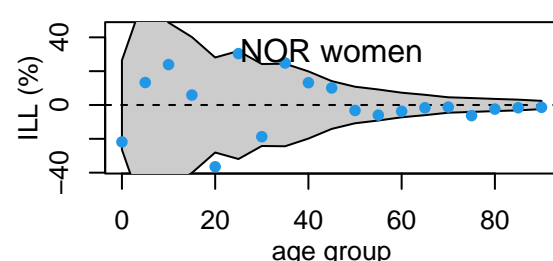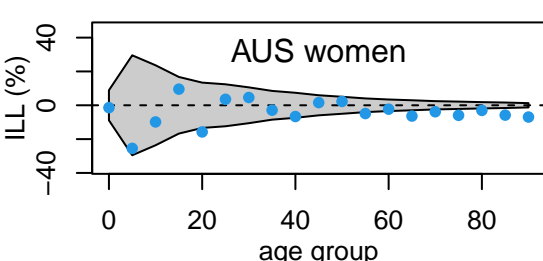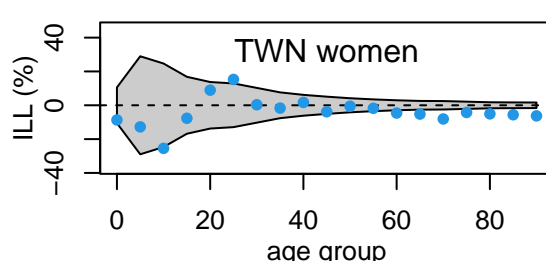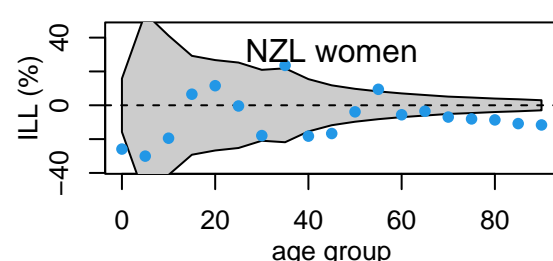

Supplement: Supplementary file 1 — Supporting Information [file BIMJ-66-e202300386-s002.zip › reproductibility/figure7s.pdf]

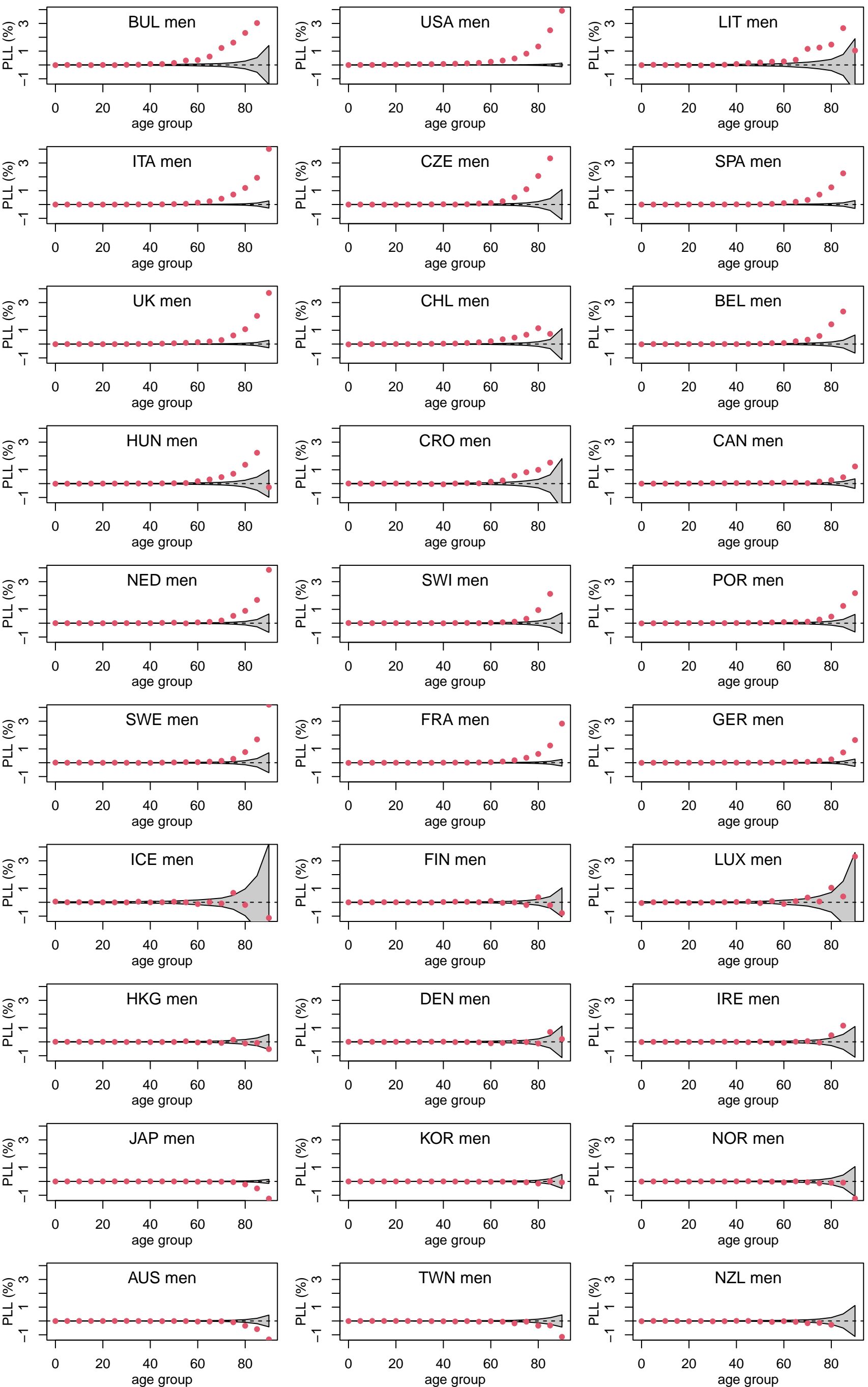

Supplement: Supplementary file 1 — Supporting Information [file BIMJ-66-e202300386-s002.zip › reproductibility/figure8s.pdf]

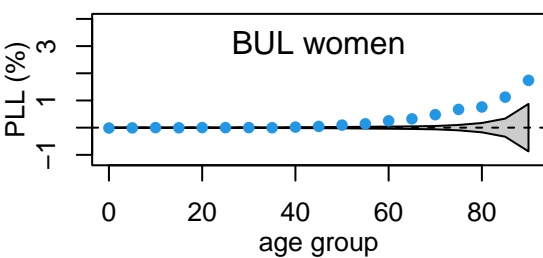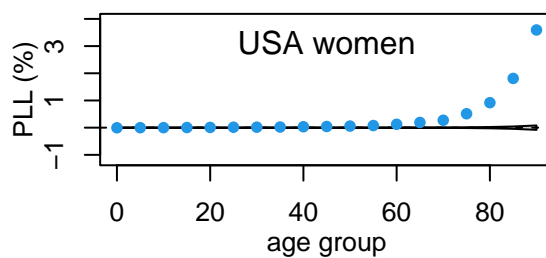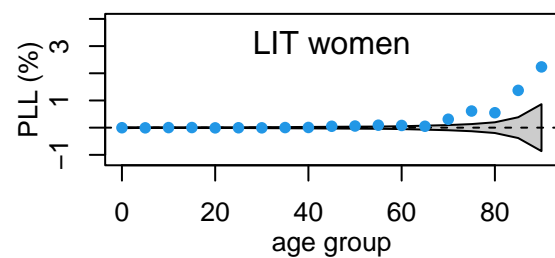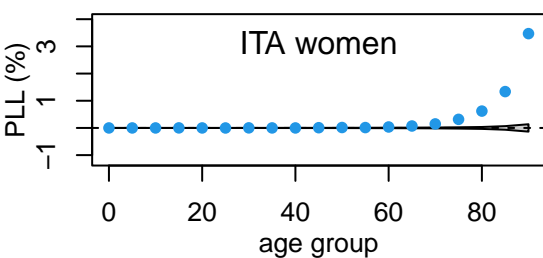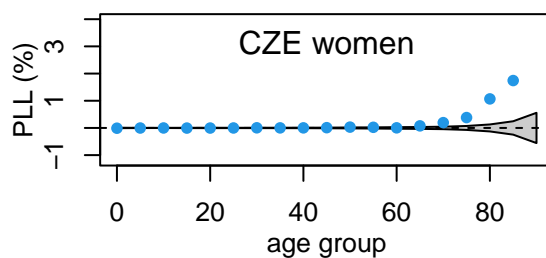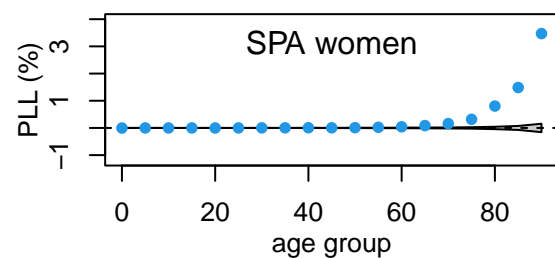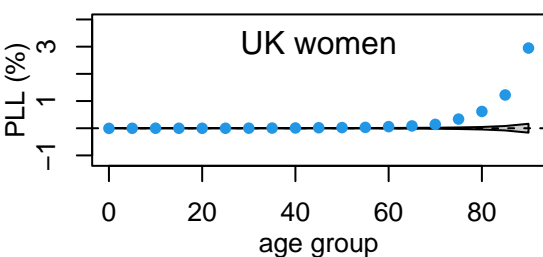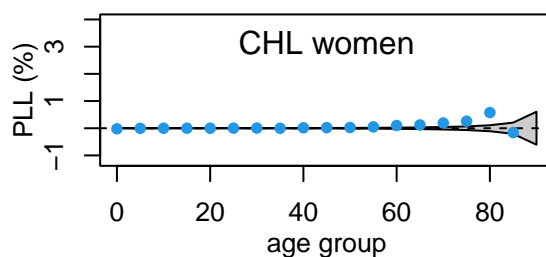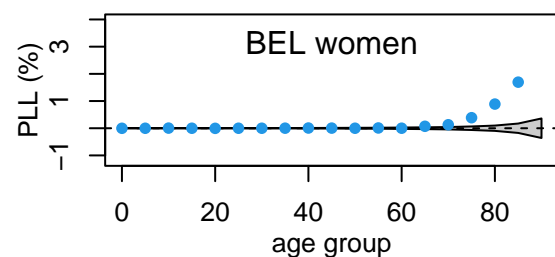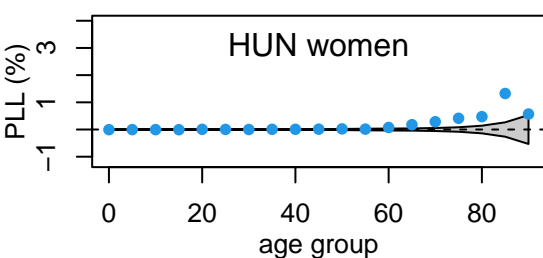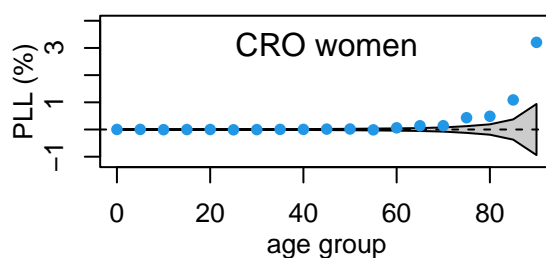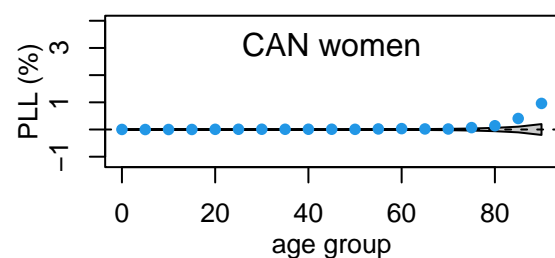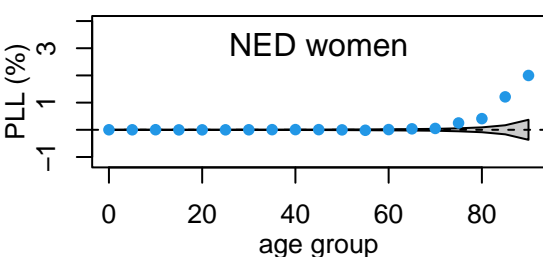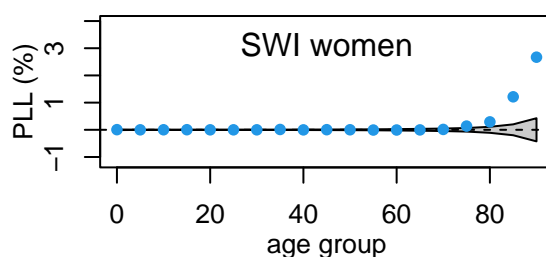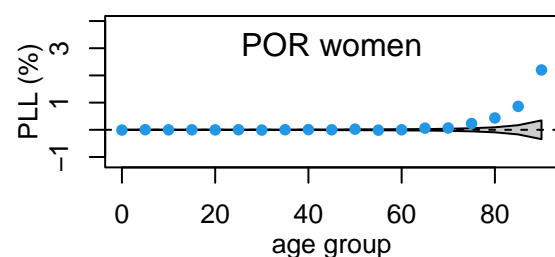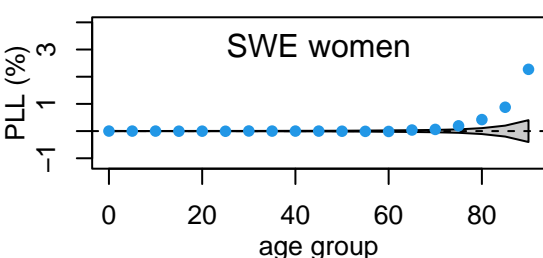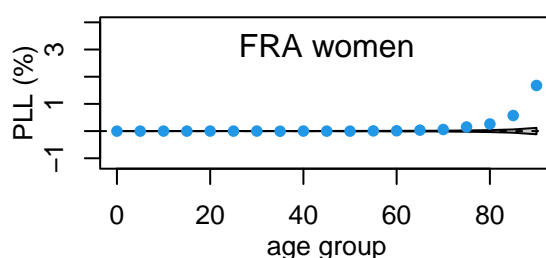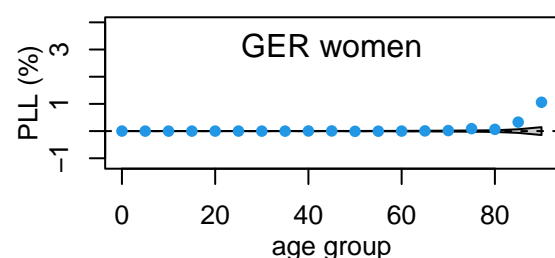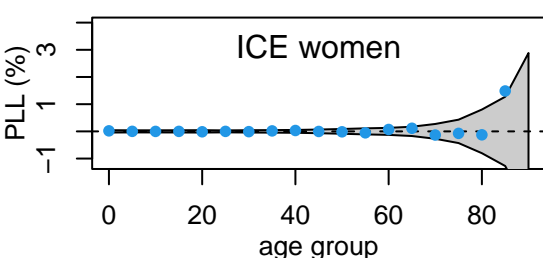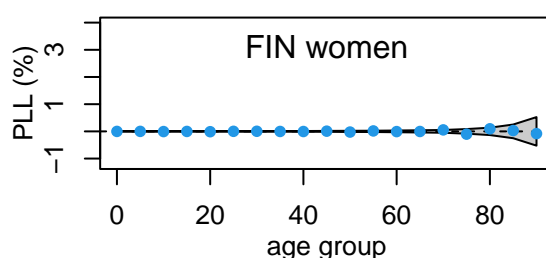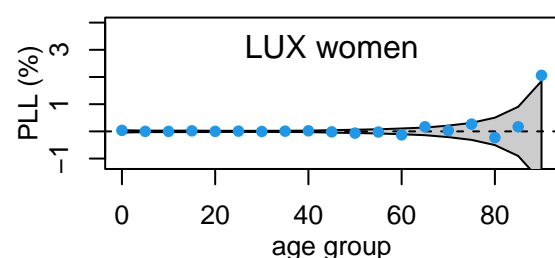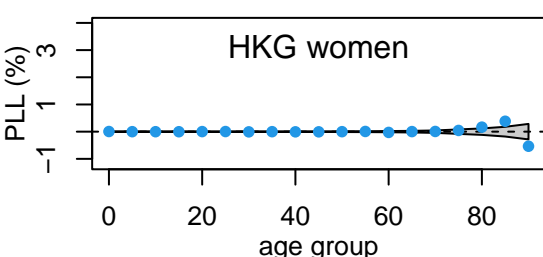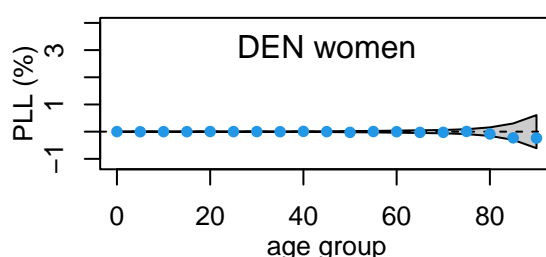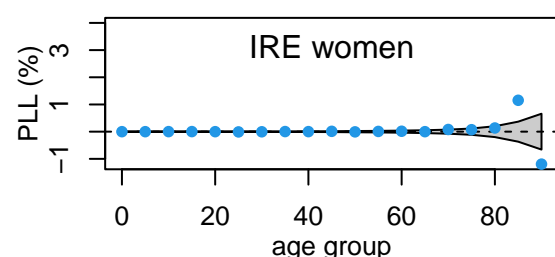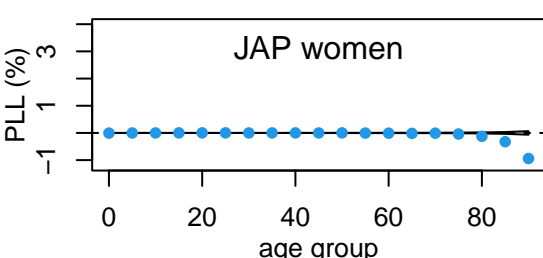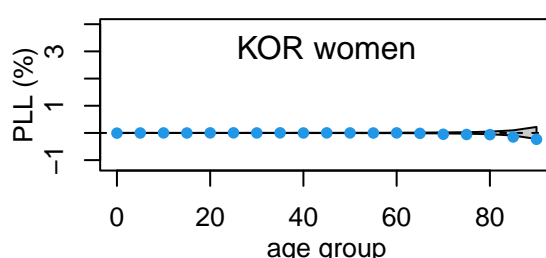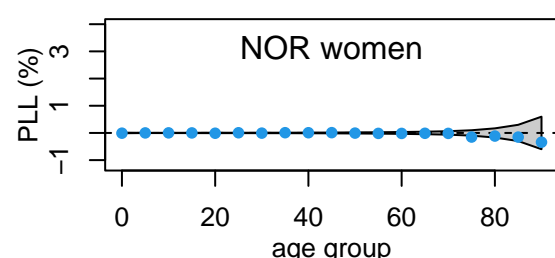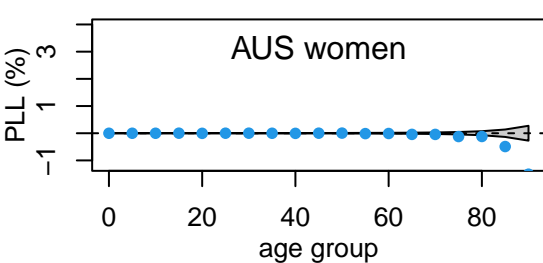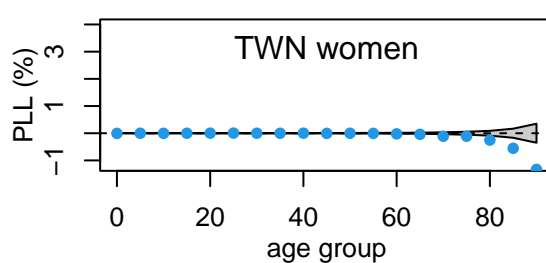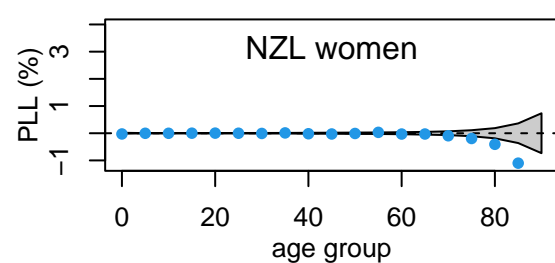

Supplement: Supplementary file 1 — Supporting Information [file BIMJ-66-e202300386-s002.zip › reproductibility/figure9s.pdf]
