# Supplementary material for: Years of Life Lost to COVID‐19 and Related Mortality Indicators: An Illustration in 30 Countries
Source: Biom J. 2024 Jul 13;66(5):e202300386. doi: 10.1002/bimj.202300386 (PMC12859533; doi:10.1002/bimj.202300386)
Supplement: Supplementary file 2 — Supporting Information [file BIMJ-66-e202300386-s001.pdf]

Years of life lost to COVID-19 and related mortality indicators: An illustration in 30 countries

Supporting Information

Valentin Rousson & Isabella Locatelli

Center for Primary Care and Public Health (Unisanté), University of Lausanne, Route de Berne 113, 1010 Lausanne, Switzerland

valentin.rousson@unisante.ch; isabella.locatelli@unisante.ch

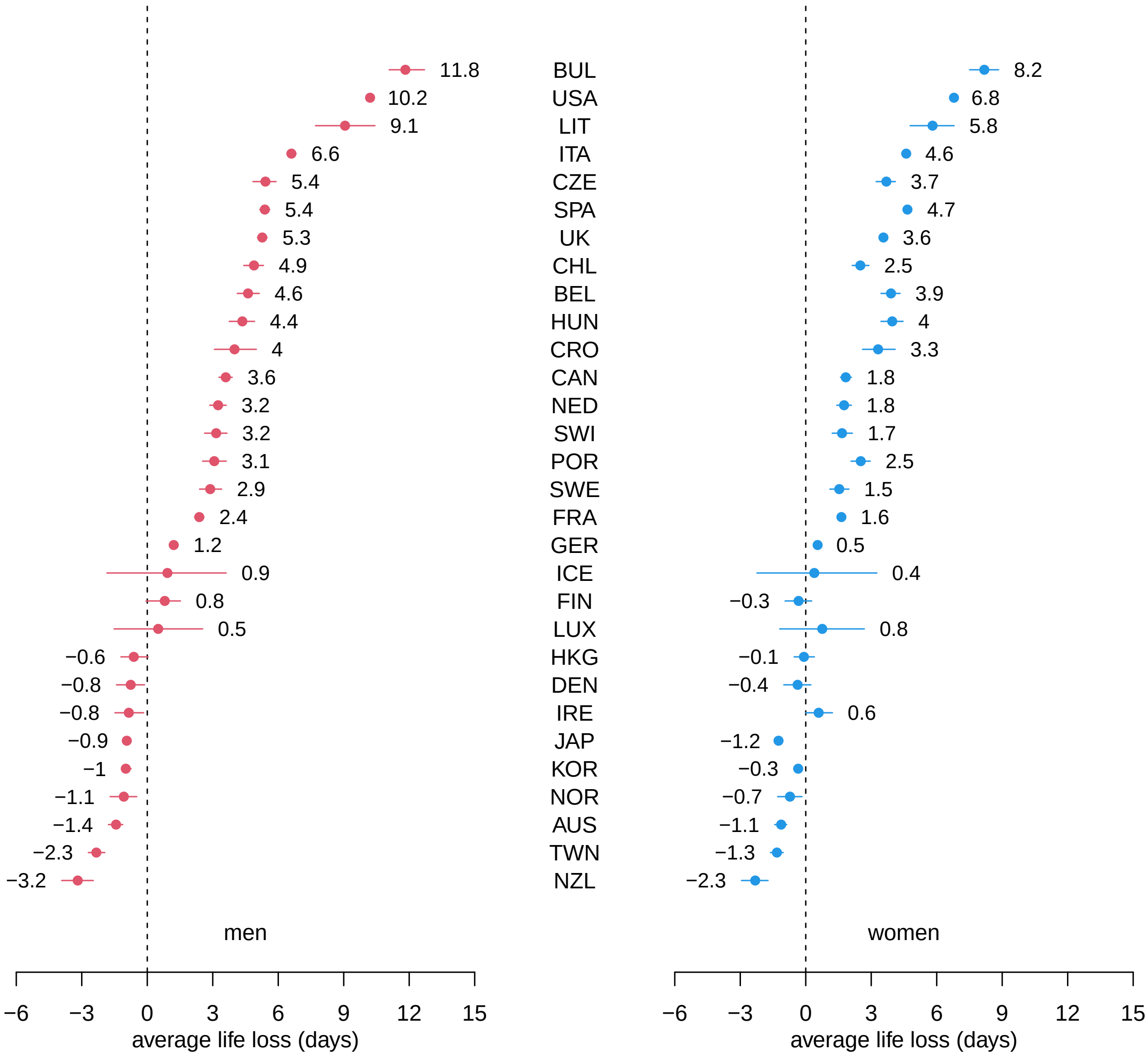

Figure 1S: Average life loss (in days) for men and women of 30 countries in 2020, together with 95% bootstrap CI.

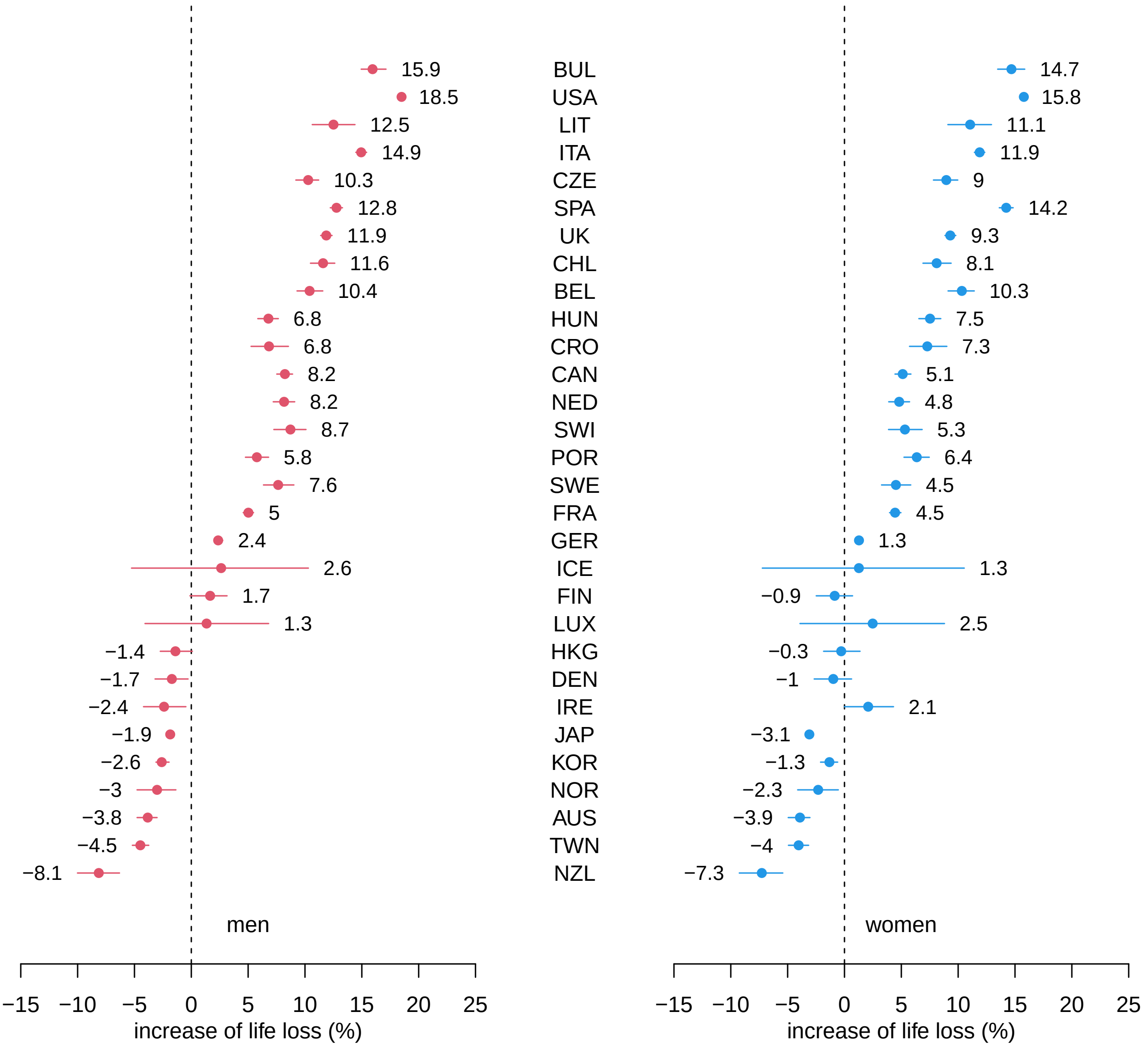

Figure 2S: Increase of life loss (in %) for men and women of 30 countries in 2020, together with 95% bootstrap CI.

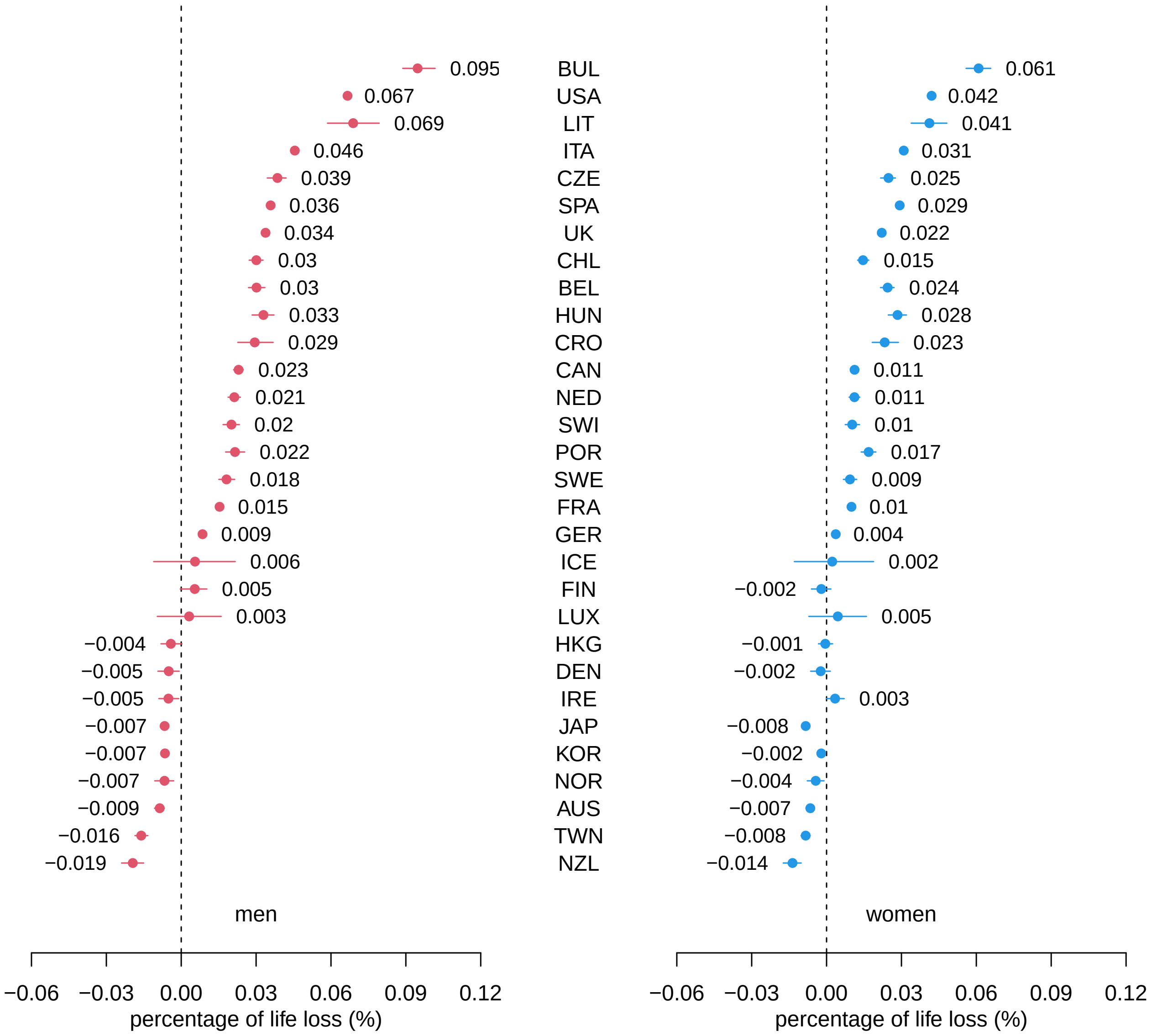

Figure 3S: Proportion of life loss (in %) for men and women of 30 countries in 2020, together with 95% bootstrap CI.

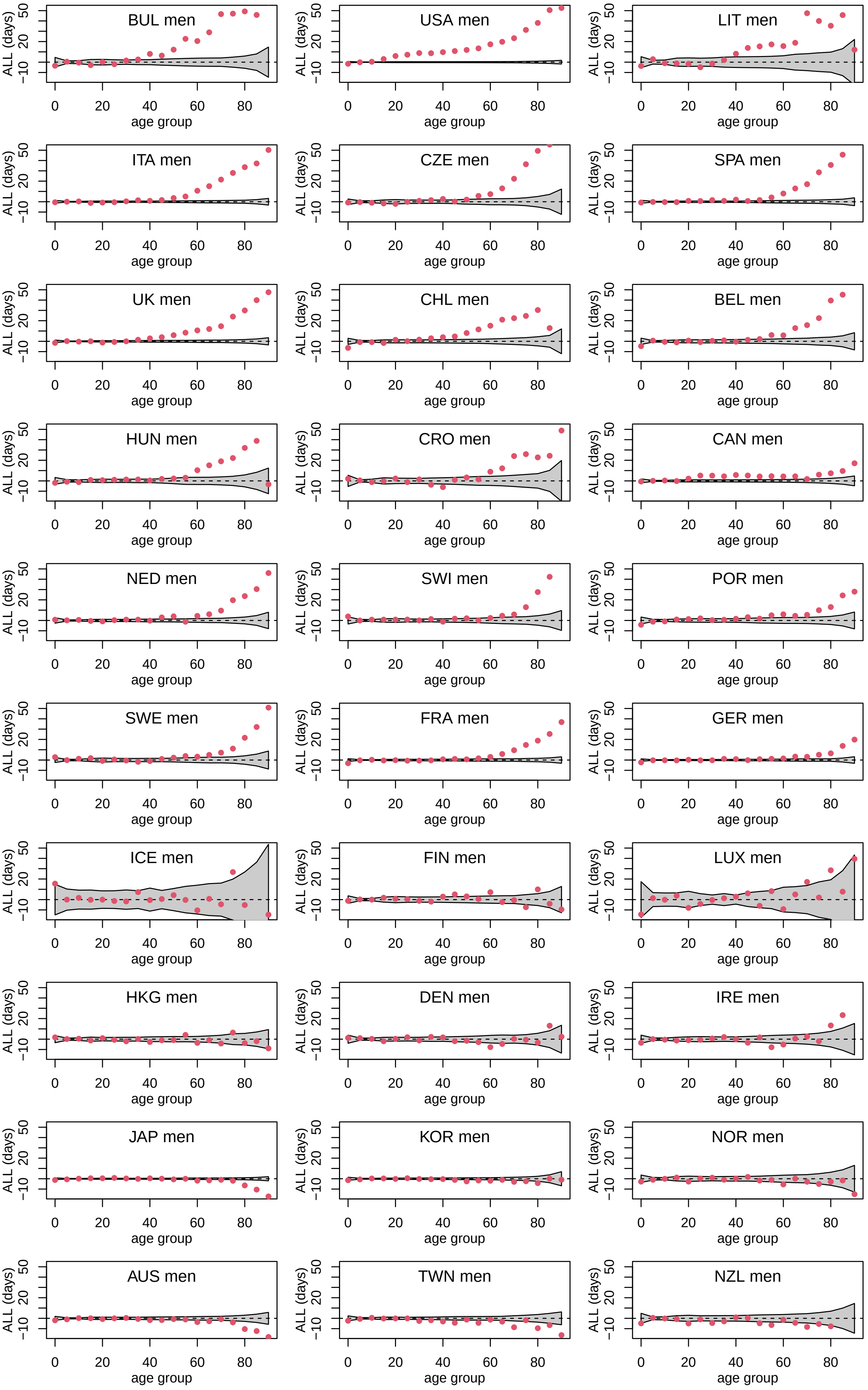

Figure 4S: Average life loss (ALL in days) calculated in 5-year age groups for men of 30 countries in 2020. Values outside the grey bands $\pm 1.96 \cdot \mathrm{Var}(\mathrm{ALL})^{1/2}$ are considered significant.

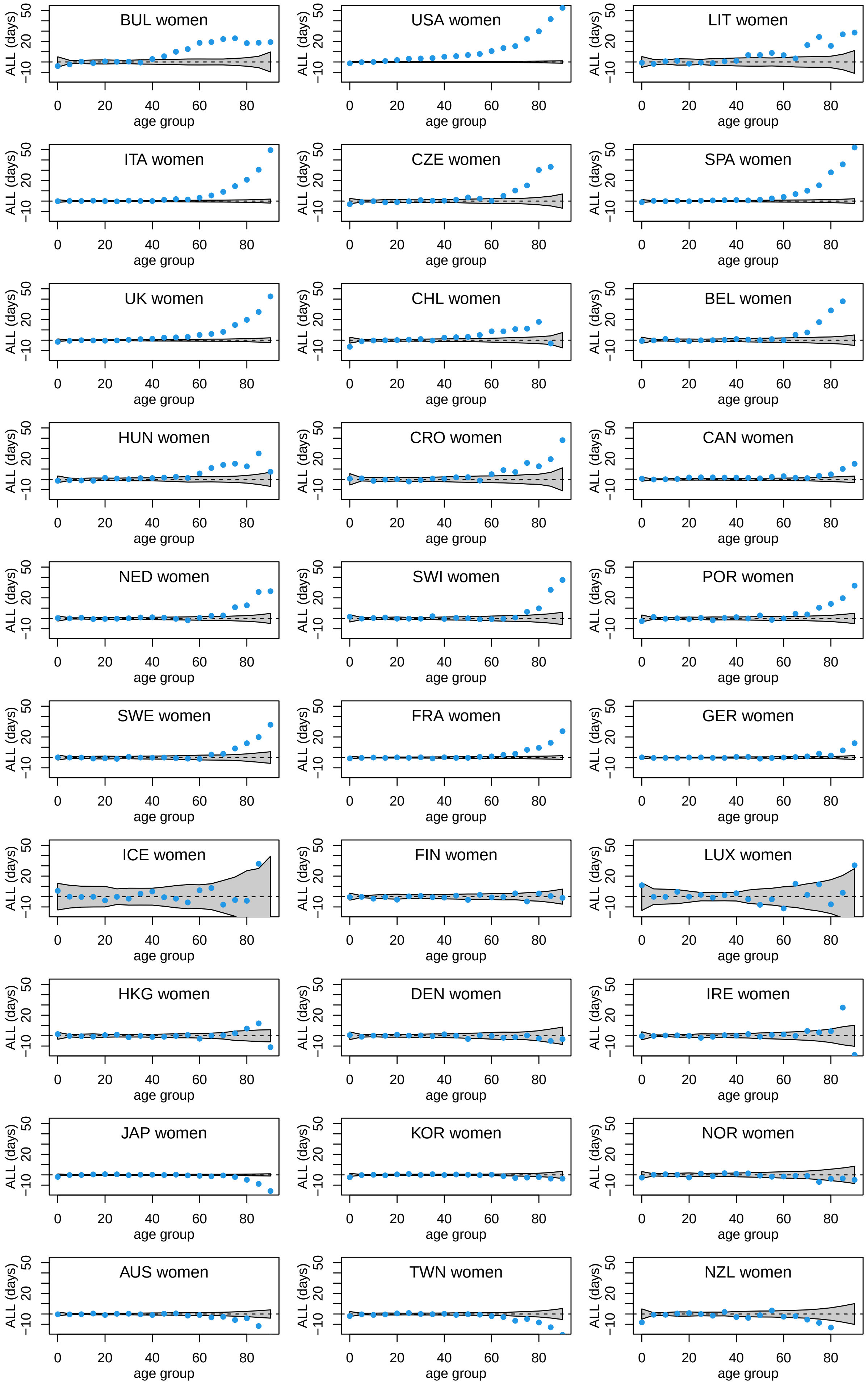

Figure 5S: Average life loss (ALL in days) calculated in 5-year age groups for women of 30 countries in 2020. Values outside the grey bands $\pm 1.96 \cdot \mathrm{Var(ALL)}^{1/2}$ are considered significant.

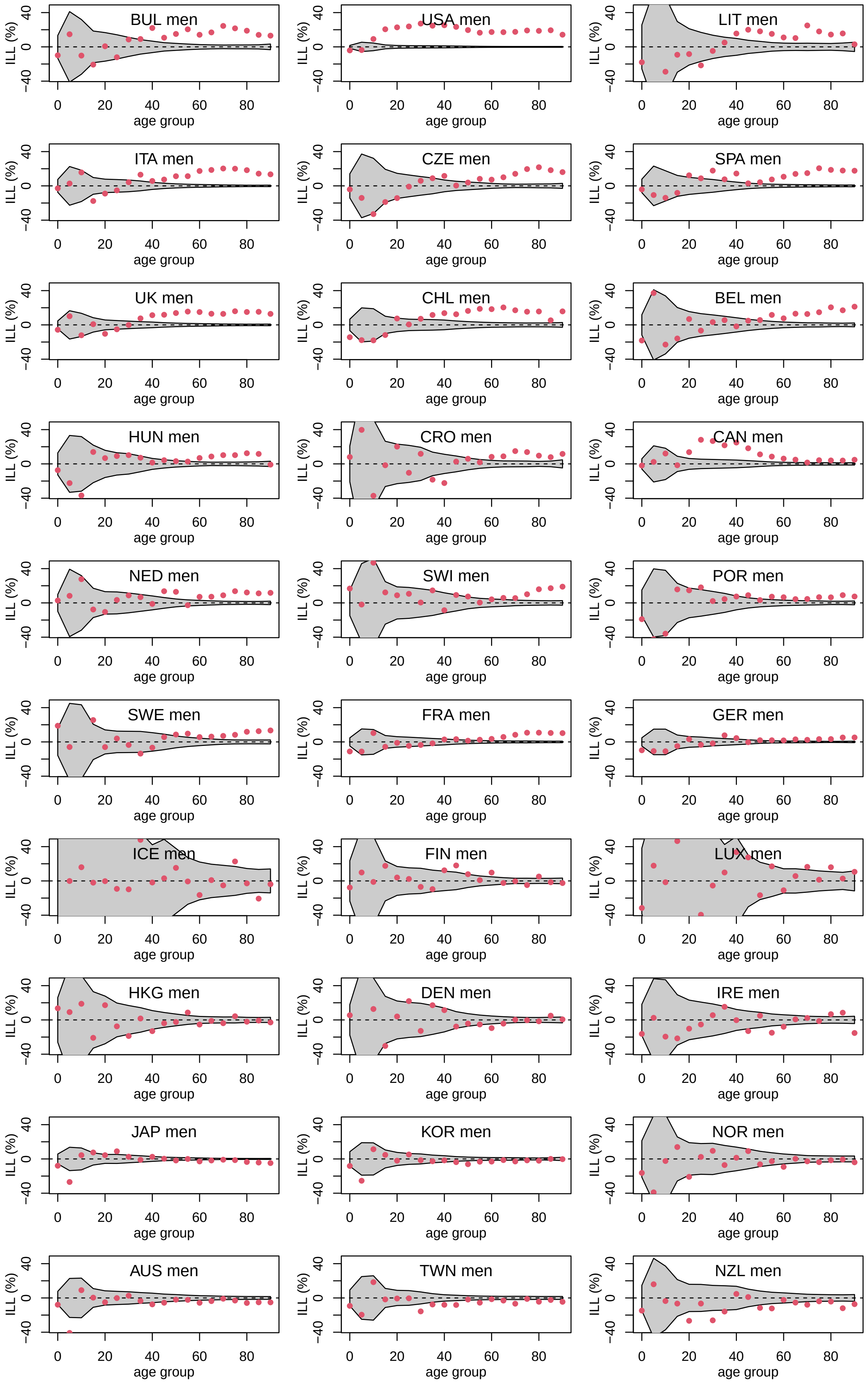

Figure 6S: Increase of life loss (ILL in %) calculated in 5-year age groups for men of 30 countries in 2020. Values outside the grey bands $\pm 1.96 \cdot \mathrm{Var(ILL)}^{1/2}$ are considered significant.

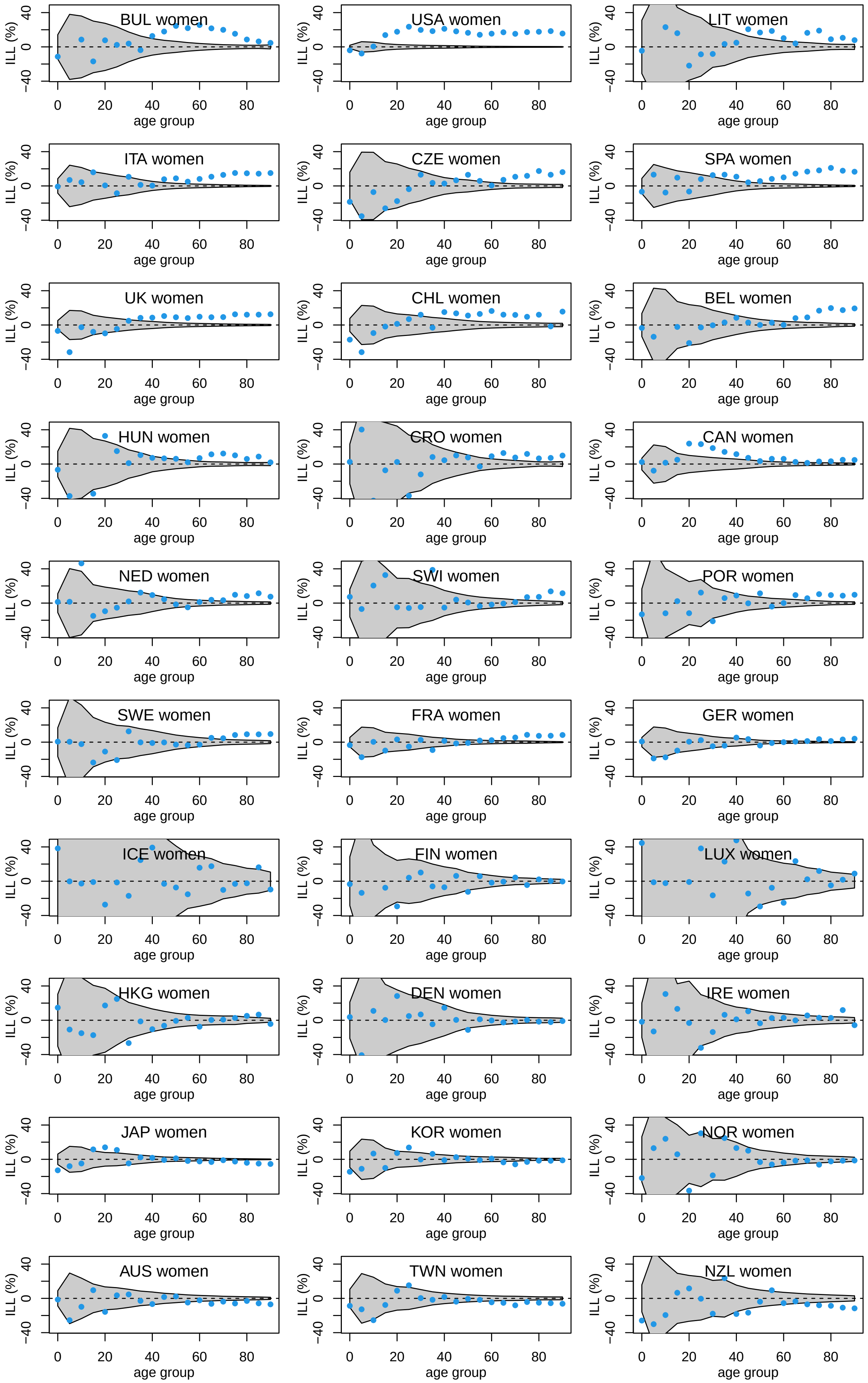

Figure 7S: Increase of life loss (ILL in %) calculated in 5-year age groups for women of 30 countries in 2020. Values outside the grey bands $\pm 1.96 \cdot \mathrm{Var(ILL)}^{1/2}$ are considered significant.

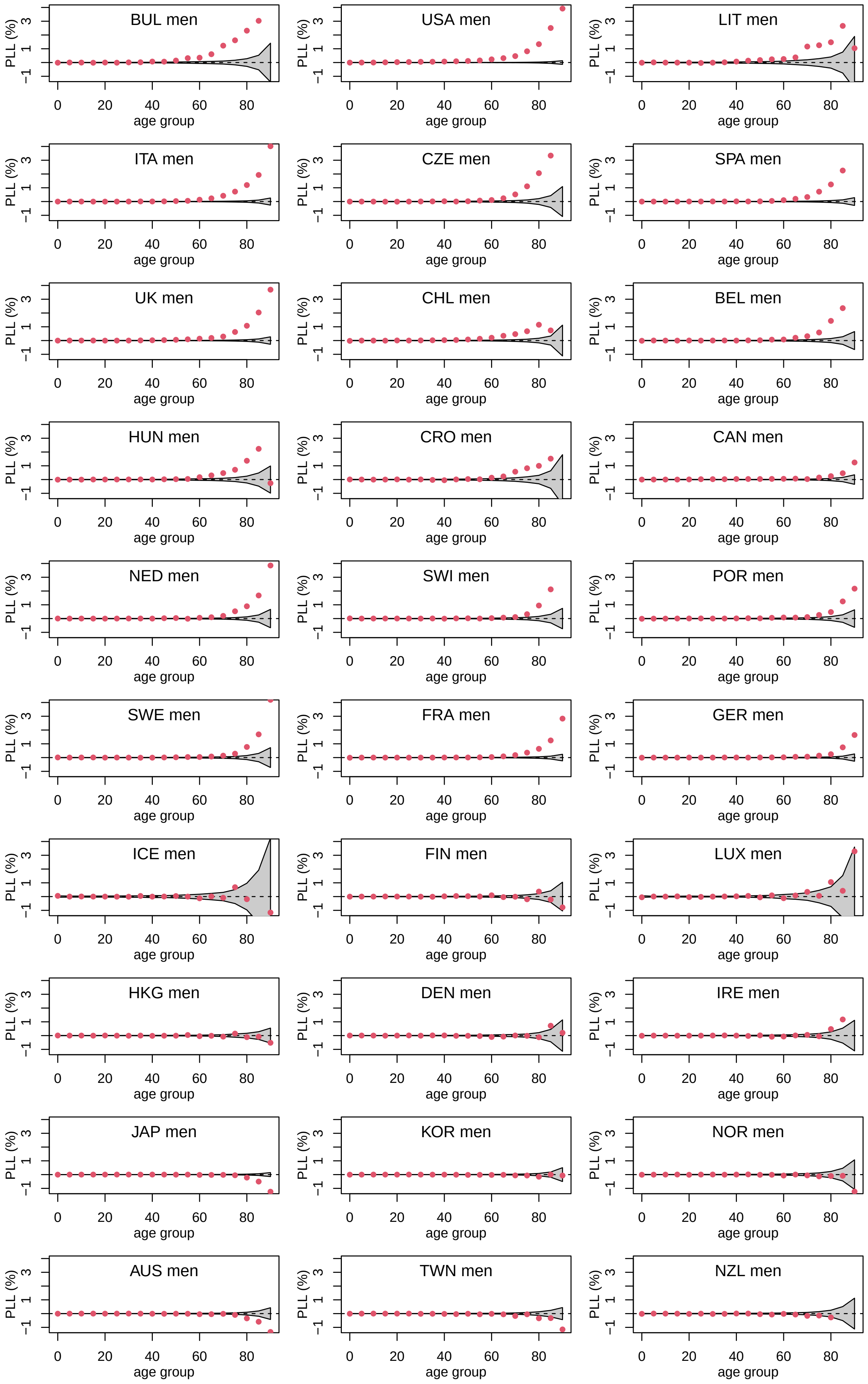

Figure 8S: Proportion of life loss (PLL in %) calculated in 5-year age groups for men of 30 countries in 2020. Values outside the grey bands $\pm 1.96 \cdot \mathrm{Var}(\mathrm{PLL})^{1/2}$ are considered significant.

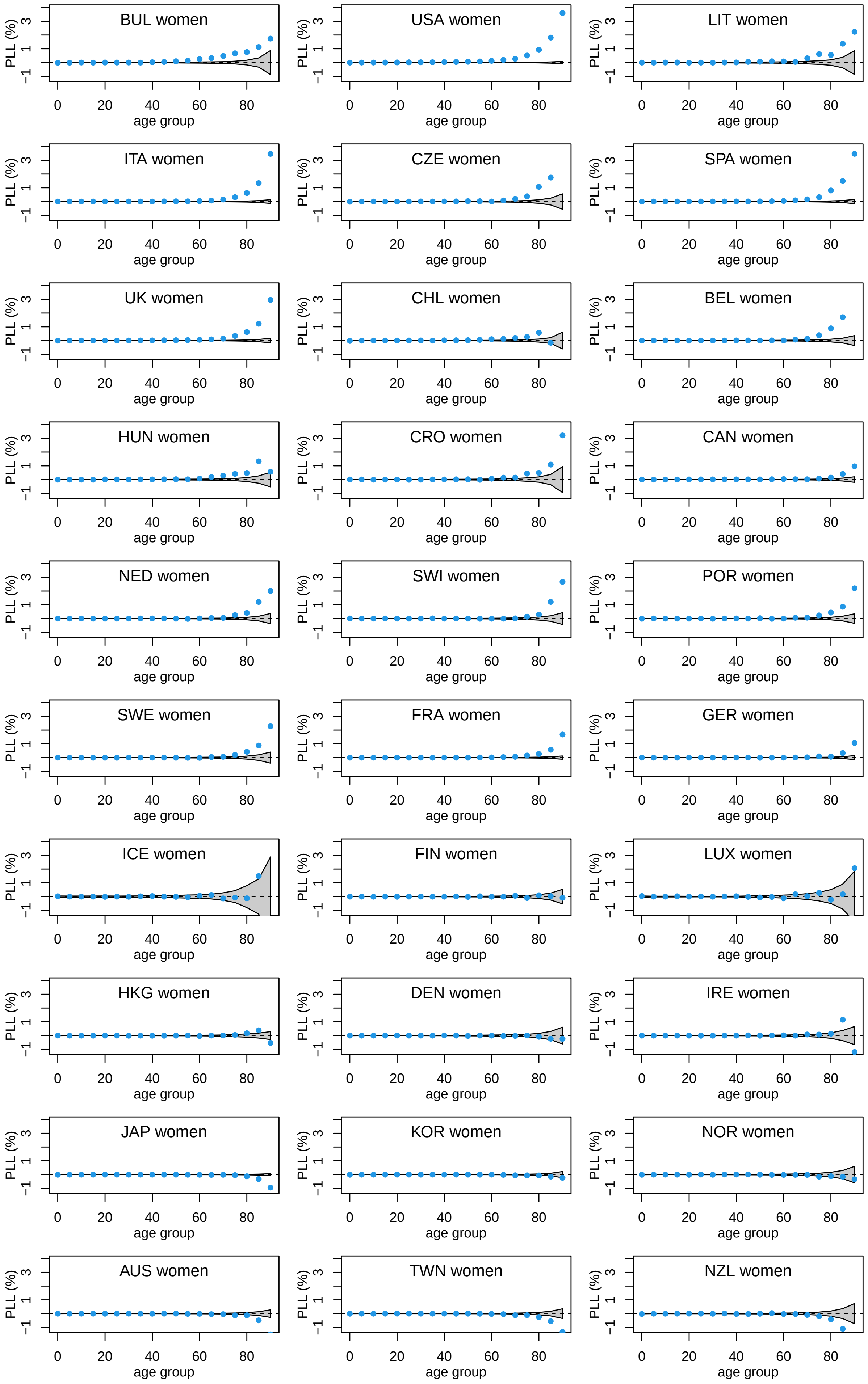

Figure 9S: Proportion of life loss (PLL in %) calculated in 5-year age groups for women of 30 countries in 2020. Values outside the grey bands $\pm 1.96 \cdot \mathrm{Var}(\mathrm{PLL})^{1/2}$ are considered significant.
